# Human milk enriched with human milk lyophilisate for feeding very low birth weight preterm infants: A preclinical experimental study focusing on fatty acid profile

**DOI:** 10.1371/journal.pone.0202794

**Published:** 2018-09-25

**Authors:** Vanessa S. Bomfim, Alceu A. Jordão, Larissa G. Alves, Francisco E. Martinez, José Simon Camelo

**Affiliations:** 1 Department of Pediatrics, Children´s Hospital, Ribeirão Preto Medical School, University of São Paulo, Ribeirão Preto, Brazil; 2 Department of Internal Medicine, Nutrition Laboratory, Ribeirão Preto Medical School, University of São Paulo, Ribeirão Preto, Brazil; 3 Human Milk Bank, Clinics Hospital, Ribeirão Preto Medical School, University of São Paulo, Ribeirão Preto, Brazil; 4 Department of Pediatrics, Neonatology, Children´s Hospital, Ribeirão Preto Medical School, University of São Paulo, Ribeirão Preto, Brazil; University of Illinois, UNITED STATES

## Abstract

**Background:**

Human milk, with essential nutrients and long chain polyunsaturated fatty acids (LC-PUFAs) such as the omega 3 and 6 fatty acids is important for development of the central nervous system and the retina in very low birth weight infants (<1,500 g). However, breast milk may not be sufficient to meet these needs. The possibility of supplementing breast milk with a lyophilisate of human milk was explored in this study. The objectives of this study were to determine the total lipid content and the lipid profile of the Human Milk on Baseline (HMB) and that of the Concentrates with the Human Milk + lyophilisate (with lyophilisate of milk in the immediate period (HMCI), at 3 months (HMC3m), and at 6 months (HMC6m) of storage).

**Methods:**

Fifty donors from the Human Milk Bank of Children’s Hospital provided consent, and donated milk samples. Macronutrient (including total lipids) quantification was performed using the MIRIS^®^ Human Milk Analyzer, and the fatty acid profile was determined by gas chromatography (CG-FID, SHIMADZU^®^).

**Results:**

There was a higher lipid concentration in HMCI relative to HMB. The concentrations of the main fatty acids (% of total) were as follows: palmitic acid (C16:0) HMB, 22.30%; HMCI, 21.46%; HMC3m, 21.54%; and HMC6m, 21.95% (p<0.01); oleic acid (C18:1n-9) HMB, 30.41%; HMCI, 30.47%; HMC3m, 30.55%; and HMC6m, 29.79% (p = 0.46); linoleic acid (C18:2n-6) HMB, 19.62%; HMCI, 19.88%; HMC3m, 19.49%; and HMC6m, 19.45% (p = 0.58); arachidonic acid (C20:4n-6) HMB, 0.35%; HMCI, 0.16%; HMC3m, 0.13%; and HMC6m, 0.15% (p<0.01); α-linolenic acid (C18:3n-3) HMB,1.32%; HMCI, 1.37%; HMC3m, 1.34%; and 1.34% HMC6m (p = 0.14); docosahexaenoic acid (C22:6n-3) HMB, 0.10%; HMCI, 0.06%; HMC3m, 0.05%; and HMC6m, 0.06% (p<0.01). There were no significant changes in the lipid profile when stored. There was no evidence of peroxidation during storage.

**Conclusions:**

Freeze-dried human milk fortified with a human milk concentrate brings potential benefits to newborns, mainly by preserving the essential nutrients present only in breast milk; however, further clinical studies are required to evaluate the safety and efficacy of the concentrate as a standard nutritional food option for very low birth weight infants.

## Introduction

The survival rate and the quality of life of preterm newborns (PTNs), especially those of very low birth weight (<1,500 g), have improved significantly due to the technical and scientific advances in preterm newborn care. The improved growth and development of these babies may also be attributed to improvements in nutritional care in the immediate postnatal period [1, 2]. The advancements in nutritional care of preterm infants aim to improve survival rates and try to simulate in-utero nutritional conditions, in order to sustain adequate neurodevelopment [3].

The nutrient intake of preterm newborns needs to be equivalent to that provided by the placenta in-utero. The latest recommendations for adequate nutritional care include increasing protein supply, improving parenteral lipid formulations, and providing mineral supplementation, in addition to feeding of human milk [2].

Human milk has a balanced nutritional composition and is the ideal food for any newborn due to its nutritional and immunological qualities. It includes essential nutrients and various types of bioactive factors, all of which contribute to the growth and development of the newborn as well as to the maturation of the gastrointestinal tract [4, 5]. In addition, breastfeeding helps in the development and strengthening of an emotional bond between mother and baby [6]. Therefore, human milk is an essential part of the diet of very low birth weight preterm infants (VLBWI) [7].

The innumerable advantages of breast milk have led to the creation of Human Milk Banks to provide food when the mother’s own milk is not available to her baby. Human milk donated to feed PTNs is pasteurized and stored till use, and this may lead to variable losses of nutrients, including lipids. [8][9] Generally, human milk is donated by mothers who have delivered full-term babies, and are at a later stage of lactation (mature milk); the composition of this mature milk differs from that of the milk from mothers who have had preterm deliveries. The milk of mothers of preterm infants contains higher concentrations of protein, sodium, zinc, and calcium than does mature milk [10] [11].

Numerous benefits of exclusive breastfeeding have been reported in the literature. However, several authors have shown that the growth rates of preterm newborn infants exclusively fed human milk were lower than those observed in the intrauterine life of full-term babies [12]. Because of their metabolic immaturity, PTNs do not accept large volumes of milk, and therefore require supplementation with hydrolyzed cow’s milk protein or with infant formulas specific for PTNs.

Human milk additives supply protein, calories, minerals, micronutrients, and electrolytes, and aid in correcting nutritional deficiencies, thus leading to improvement in growth rates of PTNs [13]. However, the high osmolality of these components may lead to adverse reactions due to unstable metabolic conditions and may increase the risk of complications associated with digestive system immaturity, such as necrotizing enterocolitis, vomiting, gastrointestinal motility problems, and sensitization to heterologous proteins [14, 15, 16].

Cristofalo et al. (2013) [17] have reported that the incidence of necrotizing enterocolitis among VLBWI who were exclusively fed formula based on cow’s milk was 21% versus that of 3% among those who were exclusively fed human milk fortified with commercial additives of human origin (Prolact^®^ + H2MF; Prolacta^®^ Bioscience); the median days with parenteral nutrition in those fed cow’s milk-based formula, and those fed fortified human milk, were 36 and 27 days, respectively.

In recent decades, special attention has been paid to the composition and the physiological aspects of the lipid fraction of human milk. The lipid portion of breast milk contributes to 40 to 55% of the total energy intake and provides essential nutrients such as linoleic acid (LA) (C18: 2ω- 6) and α-linolenic acid (LNA) (C18: 3ω-3) [18]. Long-chain polyunsaturated fatty acids (LCPUFAs) synthesized from essential fatty acids are extremely important for newborns; LCPUFAs such as docosahexaenoic acid (DHA) and arachidonic acid (ARA) are present in the phospholipids of cell membranes, and directly influence neuronal development, visual acuity, and the infant’s immune system [19, 20]. Recently, the possibility of developing a lyophilized human milk concentrate as an additive has been proposed. This is an innovative proposal, considering that in Brazil only industrialized additives manufactured from heterologous proteins (with potential to cause adverse reactions) have been used. The aim of this study was to determine the amount of total lipids, the fatty acid profile, and the nutritional stability of the Human Milk Baseline (HMB), Concentrated with the Human Milk Lyophilized in the immediate period (HMCI), and the Concentrate with Human Milk Lyophilized post-storage at 3 (HMC3m) and 6 (HMC6m) months.

## Material and methods

This preclinical experimental study is part of a project entitled “Elaboration of a Concentrate with Human Milk Lyophilisate for Feeding Very Low Birth Weight Preterm Newborns”. This project aims to formulate a concentrate with freeze-dried human milk and further to characterize its nutritional composition (levels of proteins, carbohydrates, lipids, energy, sodium, potassium, calcium, phosphorus, and magnesium, and the fatty-acid profile (particularly LCPUFA and essential fatty acids such as linoleic and alpha-linolenic acids)) and osmolarity, for use in VLBWI nutrition. This research project was carried out at the Human Milk Bank, Laboratory of Metals and Rare Diseases at Clinics Hospital, and at the Laboratory of Nutrition and Metabolism of the Department of Internal Medicine, in the Ribeirão Preto Medical School—University of São Paulo (USP).

The human milk donors were informed about the nature of the study and those who were willing to participate in the project signed a free and informed consent form; donors also underwent clinical and serological screening. There were no participants (donors) under age 18 years old.

### Ethics approval and consent to participate

This study was approved by the Human Research Ethics Committee of the Clinics Hospital, Ribeirão Preto Medical School—University of São Paulo (HREC Report No. 738.080). All participants provided written informed consent.

### Material collection, processing and quality control

Human Milk Bank donors with a lactation period greater than 15 days were given instruction about massaging and milking the breasts, and about how to withdraw the milk into a sterile, inert glass bottle provided by the Milk Bank. Considering a value of 0.36 for the standard deviation with respect to the average protein concentration (2.20 g/dL) and an absolute error value of 0.1, with a confidence level of 95%, 50 samples were required. Inclusion criteria were mature human milk with a Dornic acidity value of up to 8°D. The exclusion criteria used were a lactation period of less than 15 days and a Dornic acidity value higher than 8°D.

Donated human milk was sent to the processing room of the Human Milk Bank, where it was frozen, and a set of physical-chemical quality control procedures were carried out; a Concentrate with human milk lyophilisate was prepared, pasteurized, and was subjected to microbiological quality control procedures.

All the samples passed through the selection and classification processes recommended by the Brazilian Network of Human Milk Banks (available at: http://www.redeblh.fiocruz.br). The selection process included packaging conditions, presence of dirt, color, off-flavor parameters, and Dornic acidity. The classification process included a verification of the lactation period, Dornic acidity, and energetic content—the crematocrit [21, 8, 22].

### Obtaining the human milk concentrate

For lyophilization, 50 ml of human milk was transferred to an inert, sterile glass container and frozen at -18°C for 24 hrs. After this period, the frozen samples were placed in the vacuum chamber of a bench lyophilizer (Lyophilizer L108^®^, LioTop, São Carlos—SP—Brazil). After 72 hours, the samples were transferred from the lyophilizer to a cold chain, to be reconstituted with human milk for use.

The Concentrate with the Human Milk Lyophilisate in the immediate period (HMCI) was composed from samples that were withdrawn from the lyophilizer and reconstituted with 75 ml of the donor’s own Human Baseline Milk (HMB). These concentrates together with the Baseline Human Milk passed through the processes of pasteurization and microbiological quality control.

Lyophilization and reconstitution volumes have been decided by pilot studies which showed that this suggested proportion (75 ml of human milk: 50 ml of lyophilized human milk, in powder) reached healthier values of osmolality and optimal nutritional values. The osmolality was found around 450 mOsm/kg H_2_0, which is the cutoff level suggested by the American Academy of Pediatrics for safety of the babies. [23]

HMB and HMCI were pasteurized at 62.5°C for 30 minutes after a preheating period. After 30 minutes of thermal lethality to pathogenic bacteria, the vials were withdrawn from the bath and cooling was promoted until the human milk reached a temperature ≤ 5°C.

For the microbiological quality control check, the pasteurized HMB and HMCI samples were screened for total coliforms using bright green bile broth (50g/L; 5% w/v) contained within Durham tubes.

The concentrate with the human milk lyophilisate in the immediate period was subdivided into two other containers and stored in freezer -18°C (specifically designed for human milk storage) during a period of either 3 months (HMC3m) or 6 months (HMC6m), to evaluate nutritional stability. A total of 200 samples (50 of each group) were analyzed for total lipids (Human Milk Bank of the Children´s Hospital, Ribeirão Preto Medical School—USP) and fatty acid profile (Laboratory of Nutrition and Metabolism of the Department of Internal Medicine of the Ribeirão Preto Medical School—USP).

### Analysis of macronutrients, total lipids and fatty acid profile

Samples were homogenized using a Sonicator Miris^®^ (Miris, Uppsala, Sweden), and the macronutrient content (including total lipids) was quantified from 2 ml of sample by using the Miris Human Milk Analyzer (Miris, Uppsala, Sweden). The Miris Human Milk Analyzer performs precise and accurate analysis based on infrared transmission spectroscopy.

For the analysis of fatty acids, fat extraction was carried out using the Bligh and Dyer method [24] and by methylation with potassium hydroxide in methanol (KOH/MeOH) at 0.5 M (derivatization reaction).

The fatty acid methyl esters were determined by gas chromatography using the Shimadzu gas chromatograph (GC-2014, Shimadzu Europe, Duisburg, Germany) with a polyethylene glycol capillary column (Supelcowax 10; 30 m long, 0.25 mm in diameter, 0.25 μm film thickness; Supelco Inc., Bellefonte, PA). Helium gas was used as the drag gas at a flow rate of 1.0 ml/min. Synthetic air was used for flame ionization with detection at 280°C.

The separation of fatty acids was performed using a polyethylene glycol capillary column which was subjected to a temperature gradient. The initial column temperature was 100°C, which was held for one minute; thereafter, the temperature was increased at a rate of 13°C per minute up to 195°C, held for five minutes, and then raised at a rate of 15°C per minute up to 240°C and held at that temperature for 30 minutes. Sample injections (1 μl volume) were performed in the split mode. The injector and detector temperatures were set to 250°C. The identification of the chromatographic peaks as well as the determination of the percentage of fatty acids present in the samples (proportion in relation to the total amount of fatty acids identified) was made by comparing the retention times and the peak area of the samples with those of an external standard (Supelco 37 Component Fame Mix).

### Statistical analysis

Data are presented as mean values ± standard deviation and as minimum and maximum values. Analysis of total lipids was performed using the analysis of variance (ANOVA, general linear model) method for repeated measures with a post-hoc Bonferroni test. Due to data variability, the logarithm of exponential base (ln) of each variable was used in the analyses of lipids profile. For comparisons between the time points, linear models of mixed effects were adjusted using the PROC MIXED module of the SAS 9.3 software. Orthogonal contrasts (multiple comparisons) were estimated only in cases where the null hypothesis (no difference between the means of the time points) was rejected. In all cases, the level of significance was set at p<0.05.

## Results

Fifty mothers participated in the study. The characteristics of the mothers are summarized in Table 1. The mean age of the women was 30.4 years and the mean gestational age was 38.43 weeks. The mean weight and height of the mothers were 67.52 kg and 1.63 m, respectively. The average Body Mass Index (BMI) of the donors was 25.2 kg/m^2^. Of the donors, 60% had undergone a cesarean delivery, while 40% had undergone a normal vaginal delivery.

**Table 1:** Characteristics of the human milk donors.

Mothers (n = 50)	Mean	Standard Deviation	Minimum	Maximum
Age (years)	30.45	6.05	17	44
Weight (kg)	67.52	11.92	47	95
Height (m)	1.63	0.589	1.52	1.77
BMI (kg/m^2^)	25.2	4.39	18.1	38.1
Gestational age (weeks)	38.43	2.27	27	42

Macronutrients were determined in conjunction with total lipids by infrared transmission spectroscopy. Macro as well as micronutrients (Na, K, Ca, Mg, P, Cu, Zn) will be analyzed and discussed in a following publication. Table 2 presents data on macronutrient levels and osmolality of the human milk and milk concentrates.

**Table 2:** Macronutrient levels and osmolality of human milk and milk concentrates. Macronutrients have been analyzed using the Miris Human Milk Analyzer^®^ by infrared transmission spectroscopy. Results expressed as mean ± standard deviation.

	HMB	HMCI	HMC3m	HMC6m
Protein (g/100ml)	0.90 ± 0.48	1.47 ± 0.58	1.39 ± 0.60	1.46 ± 0.54
Carbohydrate (g/100ml)	7.08 ± 0.67	9.17 ± 0.68	9.20 ± 0.63	9.18 ± 0.64
Total solids (g/100ml)	14.29 ± 17.30	14.76 ± 1.71	14.47 ± 1.77	14.53 ± 1.71
Energy (kcal/100ml)	56.30 ± 10.50	79.96 ± 13.74	76.98 ± 13.91	77.30 ± 13.77
True protein (g/100ml)	0.75 ± 0.39	1.20 ± 0.47	1.13 ± 0.49	1.19 ± 0.43
Osmolality (mOsm/Kg)	289.48 ± 43.64	452.12 ± 59.79	456.16 ± 56.57	458.14 ± 55.66

HMB—Human Milk Baseline; HMCI: Concentrate with Human Milk Lyophilisate in the immediate period; HMC3m: Concentrate with Human Milk Lyophilisate in the 3 months period; HMC6m: Concentrate with Human Milk Lyophilisate in the 3 months period.

The mean, standard deviation, and the minimum and maximum values of the total lipids in the HMB, HMCI, HMC3m and HMC6m samples are shown in Table 3. There was a statistically significant difference between the values at baseline and those at the other time points (concentrates) (p<0.05). The HMCI, HMC3m, and HMC6m samples demonstrated higher levels of total lipids, compared to the HMB samples (p<0.05).

**Table 3:** Total lipid values of the HMB, HMCI, HMC3m, and the HMC6m samples.

Total Lipids (g/100ml)	Mean	Standard Deviation	Minimum	Maximum
HMB	2.59	1.08	0.80	5.00
HMCI	4.03	1.44	1.30	7.50
HMC3m	3.68	1.34	1.30	6.80
HMC6m	3.96	1.28	1.50	7.10

HMB: Human Milk Baseline; HMCI: Concentrated with human milk lyophilisate in the immediate period; HMC3m: Concentrated with human milk lyophilisate freeze stored for 3 months; HMC6m: Concentrated with human milk lyophilisate freeze stored for 6 months.

There were no differences in total lipid levels among the HMCI, HMC3m, and the HMC6m concentrates.

**Fig 1:**
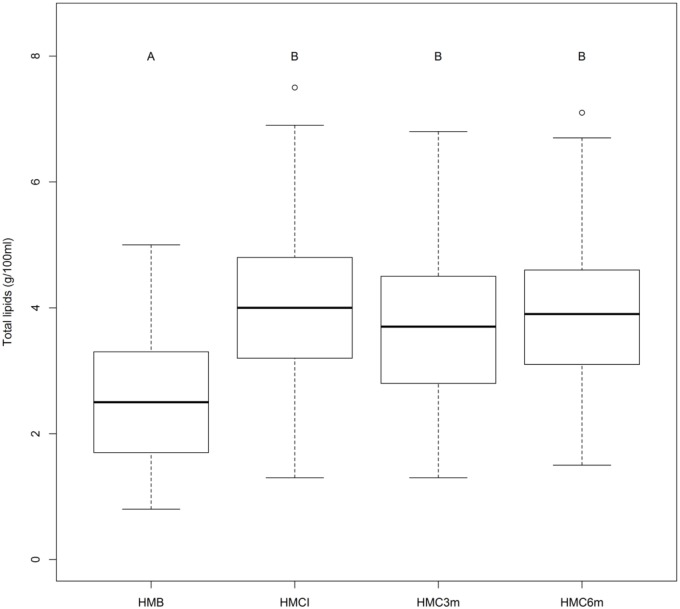
Box plot of total lipid values at HMB, HMCI, HMC3m and HMC6m. A x B: Mean values were significantly different (p<0.05).

Fig 1 presents these results.

Table 4 shows the fatty acid profile of Baseline Human Milk and that of the Concentrates. Data are expressed as percentage in relation to the total identified fatty acids.

**Table 4:** Complete fatty acid profile of the HMB, HMCI, HMC3m, and the HMC6m samples.

Fatty Acids	HMB	HMCI	HMC3m	HMC6m	p-value
C4:0 (Butyric acid)	0.01 ± 0.01^a^^,^^c^	0.01 ± 0.01^a^^,^^c^	0.01 ± 0.01^a^^,^^d^	< 0.01^b^^,^^d^	< 0.01*
C6:0 (Caproic acid)	0.05 ± 0.05^a^	0.06 ± 0.03^b^	0.06 ± 0.03^b^	0.06 ± 0.03^b^	< 0.01*
C8:0 (Caprylic acid)	0.14 ± 0.12^a^	0.18 ± 0.08^b^	0.19 ± 0.09^b^	0.20 ± 0.09^b^	< 0.01*
C10:0 (Capric acid)	1.43 ± 0.60^a^	1.64 ± 0.48^b^	1.73 ± 0.61^b^	1.72 ± 0.50^b^	< 0.01*
C11:0 (Undecylic acid)	0.09 ± 0.18^a^	0.05 ± 0.02^b^	0.04 ± 0.02^b^	0.04 ± 0.03^b^	< 0.01*
C12:0 (Lauric acid)	6.11 ± 2.30^a^	6.91 ± 1.87^b^	7.10 ± 2.27^b^	7.00 ± 1.74^b^	< 0.01*
C13:0 (Tridecanoic Acid)	0.04 ± 0.08	0.02 ± 0.01	0.03 ± 0.01	0.16 ± 0.93	0.10
C14:0 (Myristic acid)	6.51 ± 2.35^a^	6.68 ± 2.03^a^	6.82 ± 1.95^b^	6.92 ± 1.81^b^	< 0.01*
C14:1 (Myristoleic acid)	0.17 ± 0.06^a^^,^^c^	0.17 ± 0.06^a^^,^^c^	0.18 ± 0.06^b^^,^^c^	0.19 ± 0.07^b^^,^^d^	< 0.01*
C15:0 (Pentadecanoic acid)	0.26 ± 0.08	0.24 ± 0.07	0.25 ± 0.07	0.26 ± 0.07	< 0.01*
C15:1 (Cis-10-pentadecenoic acid)	0.07 ± 0.09^a^^,^^d^	0.05 ± 0.02^b^^,^^c^	0.06 ± 0.02^a^^,^^c^	0.07 ± 0.07^a^^,^^d^	< 0.01*
**C16:0 (Palmitic acid)**	**22.30 ± 2.80**^a^^,^^d^	**21.46 ± 2.23**^b^^,^^c^	**21.54 ± 2.09**^b^^,^^c^	**21.95 ± 2.06**^a^^,^^d^	< 0.01*
C16:1 (Palmitolytic acid)	1.86 ± 0.59	1.94 ± 0.52	1.93 ± 0.51	1.94 ± 0.51	0.06
C17:0 (Margaric acid)	0.29 ± 0.06^a^^,^^d^^,^^f^	0.27 ± 0.05^b^^,^^c^^,^^e^	0.27 ± 0.05^b^^,^^c^^,^^e^	0.28 ± 0.06^a^^,^^d^^,^^f^	< 0.01*
C17:1 (Cis-10-heptadecanoic acid)	0.17 ± 0.04	0.17 ± 0.03	0.17 ± 0.03	0.17 ± 0.03	0.49
C18:0 (Stearic acid)	6.44 ± 1.43^a^	6.01 ± 1.16^b^	5.92 ± 1.12^b^	6.05 ± 1.07^b^	< 0.01*
C18:1n9c (Oleic acid)	30.41 ± 4.36	30.47 ± 3.41	30.55 ± 3.56	29.79 ± 3.28	0.46
C18:2n6c (Linoleic acid)	19.62 ± 4.11	19.88 ± 3.68	19.49 ± 3.49	19.45 ± 3.40	0.58
**C18:3n6 (γ-Linoleic acid)**	**0.16 ± 0.07**	**0.16 ± 0.05**	**0.15 ± 0.04**	**0.16 ± 0.06**	**0.58**
**C18:3n3 (α-Linolenic acid)**	**1.32 ± 0.39**	**1.37 ± 0.32**	**1.34 ± 0.32**	**1.34 ± 0.30**	**0.14**
C20:0 (Arachidic acid)	0.13 ± 0.07^a^	0.11 ± 0.03^b^	0.11 ± 0.03^b^	0.12 ± 0.03^b^	< 0.01*
C20:1n9 (Gadoleic acid)	0.25 ± 0.10	0.23 ± 0.06	0.23 ± 0.06	0.23 ± 0.06	0.37
C20:2 (11,14-Eicosadienoic acid)	0.31 ± 0.10	0.30 ± 0.08	0.30 ± 0.08	0.30 ± 0,08	0.87
C20:3n6 (Dihomo-γ-linoleic acid)	0.05 ± 0.09^a^	0.03 ± 0.01^b^	0.03 ± 0.01^b^	0.03 ± 0.04^b^	0.01*
C21:0 (Heneicosanoic acid)	0.37 ± 0.11	0.38 ± 0.12	0.37 ± 0.08	0.37 ± 0.10	0.41
C20:3n3 (Eicosatrienoic acid)	0.50 ± 0.12	0.52 ± 0.11	0.52 ± 0.10	0.52 ± 0.11	0.14
**C20:4n6 (Arachidonic acid)**	**0.35 ± 0.79**^a^^,^^d^	**0.16 ± 0.08**^b^^,^^c^	**0.13 ± 0.09**^b^^,^^d^	**0.15 ± 0.10**^b^^,^^c^	< 0.01*
**C20:5n3 (Eicosapentaenoic acid)**	**0.10 ± 0.06**^a^	**0.09 ± 0.03**^a^	**0.09 ± 0.03**^b^	**0.09 ± 0.04**^b^	< 0.01*
C22:1n9 (Erucic acid)	0.04 ± 0.05^a^	0.04 ± 0.02^b^	0.03 ± 0.01^b^	0.03 ± 0.02^b^	< 0.01*
C22:2 (13,16-Docosadienoic acid)	0.04 ± 0.02	0.03 ± 0.01	0.03 ± 0.01	0.04 ± 0.01	0.14
C24:0 (Lignoseric acid)	0.12 ± 0.04	0.12 ± 0.03	0.11 ± 0.03	0.12 ± 0.03	0.12
C24:1n9 (Lignoceruleic acid)	0.16 ± 0.09^a^	0.18 ± 0.09^a^	0.17 ± 0.09^a^	0.19 ± 0.09^b^	< 0.01*
**C22:6n-3 (Docosahexaenoic acid)**	**0.10 ± 0.10**^a^	**0.06 ± 0.03**^b^	**0.05 ± 0.03**^b^	**0.06 ± 0.04**^b^	< 0.01*

Results expressed as mean ± standard deviation. HMB: Human Milk Baseline; HMCI: Concentrated with human milk lyophilisate in the immediate period; HMC3m: Concentrated with Human Milk Lyophilisate freeze stored 3 Months; HMC6m: Concentrated with Human Milk Lyophilisate freeze stored 6 Months.

*There was a significant difference between groups.

^a,b,c,d,e,f:^ Mean values with different letters are significantly different (p<0.05).

Of the total fatty acids identified in the 4 groups, oleic acid (C18:1n-9) was present in the highest proportion, with a prevalence of 30.41% in HMB, 30.47% in HMCI, 30.55% in HMC3m, and 29.79% in HMC6m samples; there was no statistical difference among the means of the groups (p = 0.46). Palmitic acid (C16: 0) comprised of 22.30% of fatty acids in HMB, 21.46% in HMCI, 21.54% in HMC3m, and 21.95% of fatty acids in HMC6m samples, with statistical difference among the means of the groups (p<0.01).

Among the PUFAs of the omega 6 class, linoleic acid (C18:2ω-6) was the most abundant, and comprised of 19.62%, 19.88%, 19.49%, and 19.45% of total fatty acids in HMB, HMCI, HMC3m, and HMC6m samples, respectively, with no statistical difference among groups (p = 0.58). The main LCPUFA of the omega 6 class, ARA (C20: 4 ω-6), comprised of 0.35%, 0.16%, 0.13%, and 0.15% of total fatty acids in HMB, HMCI, HMC3m, and HMC6m samples, respectively, with statistical difference among the means of the groups (p<0.01).

Of the total amount of identified PUFA of the omega 3 class, α-linolenic acid (C18:3ω-3) was the most abundant, and comprised of 1.32%, 1.37%, 1.34%, and 1.34% of total omega 3 PUFA content in HMB, HMCI, HMC3m, and HMC6m samples, respectively, with no statistical difference among groups (p = 0.14). The major LCPUFAs of the omega 3 class are EPA (C20:5ω-3) and DHA (C22: 6 ω-3); EPA comprised of 0.10%, 0.09%, 0.09%, and 0.09% of total amount of fatty acids in the HMB, HMCI, HMC3m, and HMC6m samples, respectively; and DHA comprised of 0.10%, 0.06%, 0.05%, and 0.06% of total fatty acids in HMB, HMCI, HMC3m, and HMC6m, samples, respectively. The content of both these LCPUFAs was statistically different among groups: EPA p<0.01 and DHA p<0.01. Tables that show values analyses ​​at different times of for all fatty acids that had showed statistical differences in levels are found presented in the supplementary material (“PublicationFiles-s1_enccompletedatabase_14febr2017.zip”).

A summary of the fatty acid composition categorized by structural class is presented in Table 5.

**Table 5:** A summary of the fatty acid composition by structural class.

Σ	HMB	HMCI	LH3m	LH6m	p-valor
SFA	44.28 ± 5.15	44.13 ± 4.46	44.55 ± 4.69	45.25 ± 4.31	0.12
MUFA	33.15 ± 4.50	33.24 ± 3.65	33.32 ± 3.82	32.62 ± 3.51	0.45
PUFA ω-6	20.18 ± 3.99	20.22 ± 3.67	19.80 ± 3.47	19.79 ± 3.36	0.32
PUFA ω-3	2.36 ± 0.43	2.38 ± 0.34	2.32 ± 0.35	2.34 ± 0.36	0.53

Results expressed in Mean ± standard deviation. SFA: Saturated fatty acids; MUFA: Monounsaturated fatty acids; PUFA ω-6: Polyunsaturated fatty acids class omega 6; PUFA ω-3: Polyunsaturated fatty acids class omega 3.

It should be noted that the lipid profile found reported here is representative only of this study population alone.

## Discussion

The present study reports the development of a human milk concentrate formulated using a lyophilisate obtained from mature human milk, and presents data about the macronutrient levels of the concentrate, focusing on the lipid profile. Data presented here show that the composition and osmolality of the formulation are within the range that is accepted as safe and adequate for VLBW infants. In addition, a detailed discussion of the lipid profile of the concentrate has been included.

Although breast milk is the best option for feeding preterm infants, the nutrient content of breast milk is not always sufficient to meet the needs of VLBW infants. Due to the high nutritional variation inherent among breast milk from different women, it is challenging to develop standard fortification formulations; in addition, heterologous protein-based additives in milk supplements are widely known to increase the occurrence of food intolerance and necrotizing enterocolitis [25, 26].

There is a growing trend of bedside analysis of human milk in Neonatal Intensive Care Units, in order to personalize enteral nutrition in VLBW infants. Infrared human milk analyzers allow for rapid analysis of breast milk and provide information about the macronutrient content; this facilitates the development of individualized fortification formulations for each preterm newborn. A well-calibrated, human milk analyzer can thus be very useful [27, 28, 29].

To improve the growth and development rates of VLBW infants, recent research has focused on new fortification strategies for human milk to optimize the intake of macro and essential micronutrients by these infants [25]. Thus, to fortify human milk with a higher concentration of nutrients, addition of human milk lyophilisates to the milk is being explored.

In the present study, analysis of lipid profiles showed that the concentrate with human milk lyophilisate (HMCI) had a higher lipid content that did the human milk baseline (HMB), indicating that HMCI may be a better source of energy than HMB.

In this study, we characterized the fatty acid profile and identified and quantified the levels of component fatty acids in human milk. To the best of our knowledge, the analysis of the fatty acid profile of samples concentrated with lyophilized human milk is unprecedented. In a study carried out at the Federal University of Mato Grosso do Sul, Grance et al. (2015) have formulated a homologous additive with a partial removal of lactose by precipitation, and have analyzed the macronutrient and electrolyte content of the formulation; however, the fatty acids profile of this formulation was not reported. The lipid content of the HMB and HMCI samples in the present study was higher than that of the formulations reported by Grance et al. (2015). The lipid content as reported by present study was 2.59 g/100 ml in HMB and 4.03 g/100 ml in HMCI samples, while the lipid content reported by Grance et al. was 1.49 g/100 ml in human milk without a homologous additive, and 2.91 g/100 ml in human milk with a homologous additive [30].

In 2012, Thomaz et al. made a comparison of levels of lipids and other macronutrients/electrolytes between human milk with homologous additives (liquid and lyophilized) and that with a commercial supplement (FM85—Nestlé). The results obtained showed that the lipid content of human milk with either liquid or lyophilized additives was 3.75 g/100ml, and that of human milk with the FM85—Nestlé additive was 3.73 g/100 ml milk, with no statistical difference among the formulations [31].

Human milk fortified with medium chain triglycerides (MCTs, Liquigen Danone Nederland) has been reported to contain 4.20 g/100ml of lipids, while human milk fortified with MCTs plus Enfamil human milk replacer (Mead Johnson) was found to have 5.09 g/100ml of lipids [32].

McLeod et al analyzed human milk fortified with a specific energy supplement (Duocal; SHS International Limited) and reported a lipid content of 4.3 g/100 ml [33].

As mentioned above, several studies have reported total lipid content of human milk enriched with different additives, similar to the present study. The nutritional stability of concentrates containing human milk lyophilisates in a post-storage period of 3 and 6 months was also analyzed with reference to lipid levels; our results revealed a lipid content of 3.68 g/100 ml and 3.96 g/100 ml in HMC3m and HMC6m samples, respectively, with no statistical difference between the two values (p>0.05). The statistical differences among HMCI and stored concentrates at 3 (p = 0.07) and 6 months (p = 0.05) were also not significant (Table 3). These data suggest that frozen storage conditions do not induce lipid losses.

Palmitic acid (C16:0) is the most abundant SFA, which is synthesized endogenously as well as obtained from the diet, and plays vital roles in the human body. Although often implicated in the pathophysiology of several adult degenerative diseases, palmitic acid is considered to be an essential part of cell membranes, and plays a crucial role in trafficking of molecules across the membranes as well as in extracellular lipid secretion, protein palmitoylation, and cell signaling [34, 35, 36].

The lipid composition of mature human milk (HMB) reported in this study (44.30% SFA, 22.30% palmitic acid, 20.20% ω-6 PUFA, and 2.40% ω-3PUFA) is in close agreement with that reported in literature. Delplanque et al. (2015) reported that mature human milk typically contains from 34 to 47% of SFA (especially palmitic acid (C16:0), which is between 17 and 25% of the fatty acid content), 12 to 26% of omega 6 PUFA, and between 0.8 and 3.6% of omega 3 PUFA. The proportion of SFA at 6 months of storage (HMC6m) was slightly higher than that at other time points, though it was within the accepted normal range [37].

LCPUFAs are present in human milk and are essential nutrients for the newborn, since they are critical components of the brain (especially the omega 3 LCPUFAs). Omega 3 fatty acids play a fundamental role in neuronal growth, myelination and dendritic tree formation, signal transduction and excitability of neural membranes, and in the expression of genes that regulate cell differentiation and its growth [38].

The structure of triglycerides present in breast milk may be considered as unique [39, 40, 36]. LCPUFAs in breast milk are linked to both triglycerides (80–90%) and phospholipids (10–20%). Approximately 110 mg/day of DHA are transferred daily through breast milk, and 20% of this DHA is present in the form of phospholipids, which are better absorbed and incorporated by the newborn [41, 19].

DHA, the most important omega 3 LCPUFA, plays vital roles in the structure and function of the retina and the brain, and is crucial for proper myelination and brain synapse formation during fetal life and childhood [42]. ARA is a key component of cell membranes and is one of the most abundant fatty acids in the brain, helping to maintain fluidity of the cell membrane of the hippocampus, protecting against oxidative stress, and activating a key protein (Syntaxin-3) involved in the growth and repair of neurons [43, 44, 45].

Although relatively low concentrations of LCPUFAs are found in human milk, some studies of Brazilian women demonstrate a deficient nutritional status pertaining to DHA, probably as a direct consequence of a diet deficient in fish and algae oils, and due to the high consumption of vegetable oils [46, 20].

Dietary intake of seafood is extremely variable and explains the broad range of DHA levels in human milk. Some studies indicate that the concentration of DHA in breast milk is between 0.1 and 1.0% of total fatty acids, although the mean concentration is about 0.33% [47, 48, 19, 49].

Women in populations living in regions near the coast or lakes may have higher levels of DHA in their breast milk due to inclusion of LCPUFA-rich fish in the diet; a previous study has found that compared to the normal world values, higher DHA and ARA concentrations of are present in the breast milk of women (0.73% of DHA and 0.69% of ARA in mature milk) living near Lake Malawi (Republic of Malawi, East Africa) [50].

The DHA levels found in HMB in the present study (0.10%) were lower than those reported in studies from other countries: Portugal, 0.43% [51]; Germany, 0.35% [52]; Italy, 0.26% [53]; Hungary, 0.23% [54]; Taiwan, 0.98% [55]; Israel and the Netherlands, 0.17% [56, 57]; China, 0.30% [58]; South Korea, 0.67% [59]; and Spain, 0.21% [60]. In Brazil, a recent study in Rio de Janeiro reported a DHA content of 0.17% in milk sourced from breastfeeding adolescents [61]. Nishimura et al. (2013) evaluated the fatty acid composition of mature human milk from women residing in Ribeirão Preto/São Paulo (far from the Brazilian coast), and found that the DHA content in human milk was among the lowest reported so far (0.09%) [20].

Overall, human breast milk in developed countries has been found to be low in nutrients such as vitamin D, iodine, iron, and vitamin K. Additional nutrient deficiencies have been documented in resource-poor countries, such as those of vitamin A, vitamin B_12_, zinc, and vitamin B_1_/thiamin. Given these findings, some authors have described breast milk as "conditionally perfect" [62]. Fortifying banked human milk with a human milk lyophilisate could help to overcome these potential deficiencies.

When each fatty acid is analyzed separately, it can be seen a decrease in the relative proportions along the different times, as observed with ARA levels, main omega 6 LCPUFA (HMB, 0.35%), decreasing its relative percentage levels at the concentrates, HMCI 0.16%, HMC3m 0.13% and HMC6m 0.15%. The levels of the main omega 3 fatty acids, EPA e DHA suffered modifications as well. EPA decreased from 0.10% at HMB to 0.09% at the concentrates (statistically different baseline *versus* concentrates). DHA decreased from 0.10% (HMB) to 0.06% (HMCI), 0.05% (HMC3m) and 0.06% (HMC6m). These results suggest a possible oxidation process of these fatty acids. However, the summation of the omega 6 and 3 fatty acids did not show differences along the time, (p = 0.32) e (p = 0.53), respectively. (Table 5) Furthermore, even with these specific percentage drops, total lipids content increased in the concentrates with the addition of the lyophilisate to the human milk baseline, offering more lipids to VLBWI anyway, in order to reach their energetic needs.

The manipulation and storage of the human milk may induce these specific modifications in some fatty acids. Notwithstanding, when the fatty acids are analyzed by the sum of structural classes, they do not show differences, and was possible to keep the human milk free of contamination, turning to a viable and safe product for consumption.

In general, pasteurization of donated human milk is effective in eliminating the risk of transmission of infectious agents, but this process may affect nutritional and immunological components adversely (although it is difficult to quantify the level of degradation) [63, 64]. A study performed by Vieira et al. (2011) showed a 5.5% reduction in lipid concentrations in human milk due to pasteurization [65], while a different study reported no significant differences in nutritional status after the pasteurization process [66].

Despite the controversy, when it is not possible for the mother to provide her own breast milk to the baby, the bank’s pasteurized human milk is the next best option. Data from clinical practice show that many properties of human milk are preserved after pasteurization, and hence pasteurized milk provides several advantages over formula milk. Overall, new technologies which are being applied for preserving bioactive and nutritional components of other foods, such as high pressure processing, are being studied as possible alternatives for thermal pasteurization [67, 64, 68].

Studies show that the lyophilization process can increase the shelf life of some foods by preventing microbial growth and slowing the oxidation of fatty acids. Compared to simple freezing (usually performed for preserving human milk), lyophilization of human milk is reported to better preserve nutritional characteristics and the integrity of several immune components while preventing oxidative deterioration [69, 70, 71].

Lozano et al. analyzed the composition of fatty acids and the antioxidant capacity of samples of lyophilized human milk at different time points up to 3 months at temperatures of 4°C and 40°C, and showed that there were no statistically significant differences in fatty acid profiles (including ARA and DHA) at the two storage temperatures. Antioxidant capacity remained constant in the samples stored at 4°C, with loss only in the samples stored at 40°C after 5 days of storage. The study concluded that the stability after storage of lyophilized human milk was higher than that reported for frozen or fresh milk [70].

In another study, human milk samples were studied after three different treatments: freezing and storage for 6 months at -80°C, lyophilization and storage for 6 months at 4°C, and lyophilization and storage for 6 months at -80°C. The levels of macronutrients, polyphenols, and oxidation markers were analyzed. The authors concluded that an adequate conservation of the analyzed components was observed in all the lyophilized samples. Freeze-drying may thus preserve the nutritional properties and oxidative integrity of milk, and Human Milk Banks may benefit from implementing this easy-to-perform treatment [71].

The present study performed pilot analyses in order to observe occurrence of lipid peroxidation and antioxidant capacity of the samples during storage, but it was not able to figure out a definitive conclusion. The interpretation was very difficult, considering the existence of several factors present into the human milk, which potentially can avoid lipid peroxidation, such as lipid soluble vitamins and antioxidant enzymes not assessed in this study [72, 73]. We decided to not include these results related to the peroxidation in the present study, due to the large variability, however, it was used an indirect method described by Pamplona et al, 1998, Peroxibility Index (PI), in order to estimate lipid peroxidation during storage, calculated based upon the degree of unsaturation of fatty acids. The formula utilized to calculate this index is described as follows: *PI = (% monoenoic x 0*,*025) + (% dienoic x 1) + (% trienoic x 2) + (% tetraenoic x 4) + (pentaenoic x 6) + (hexaenoic x 8)* [74]. Values of PI for the samples HMB, HMCI, HMC3m and HMC6m, (respectively 26,9, 26,8, 26,2 e 26,3) indicate a subtle decrease on the proportions of unsaturation along the time. This small difference among peroxibility indices may be associated to a probable oxidation of ARA, EPA e DHA, but it does not seem to be a relevant process.

The nutritional deficiencies existing in pregnancy are generally reflected in the nutritional profile of breast milk and may lead to similar deficiencies in the infant since the lactating mother will not be able to pass the deficient nutrient to the baby [62]. A limitation of this study was that we did not use a questionnaire to evaluate the nutritional status of the donors during the pre-gestational, gestational, and post-gestational periods.

## Conclusion

The production of a concentrate with freeze-dried human milk brings great benefits to the newborns by offering high quality nutrients and preserving the nutrients present only in breast milk. The concentrate fortified with human milk lyophilisate has good nutritional stability and retains the original fatty acid profile during 6 months of frozen storage.

Further clinical trials are necessary to evaluate the performance of the human milk concentrate as a feed for very low birth weight infants; the safety and tolerability of this formulation needs to be evaluated as well.

## Supporting information

S1 File Descriptive statistics. (RTF)

S2 File Final results lipidic profile. (XLSX)

S3 File Final results Miris analysis. (XLSX)

S4 File Results statistics variance. (XLSX)

S5 File Statistical analysis. (XLSX)

S6 File Qualification 08.02.17 Vanessa S Bomfim. (DOCX)

S1 Fig Palmitic acid box plot. (PNG)

S2 Fig Oleic acid box plot. (PNG)

S3 Fig Linoleic acid box plot. (PNG)

S4 Fig Arachidonic acid box plot. (PNG)

S5 Fig Linolenic acid box plot. (PNG)

S6 Fig Docosahexaenoic acid box plot. (PNG)

S1 Table Comparative values of saturated fatty acids (SFA) at different analysis times. (PDF)

S2 Table Comparative values of monounsaturated fatty acids (MUFA) at different times of analysis. (PDF)

S3 Table Comparative values of polyunsaturated fatty acids (PUFA) at different analysis times. (PDF)

## Abbreviations

ARA: Arachidonic acid
BMI: Body Mass Index
DHA: Docosahexaenoic acid
EPA: Eicosapentaenoic acid
HMB: Human Milk on Baseline
HMC3m: Concentrated with lyophilized human milk in the period of 3 months
HMC6m: Concentrated with lyophilized human milk in the period of 6 months
HMCI: Concentrate with lyophilized human milk in the immediate period
LA: Linoleic acid
LCPUFA: Long chain polyunsaturated fatty acids
LNA: α-Linolenic acid
MCTs: medium chain triglycerides
PTN: preterm newborn
USP: University of São Paulo
VLBWI: Very Low Birth Weight Infants
