# Supplementary material for: Human milk enriched with human milk lyophilisate for feeding very low birth weight preterm infants: A preclinical experimental study focusing on fatty acid profile
Source: PLoS One. 2018 Sep 25;13(9):e0202794. doi: 10.1371/journal.pone.0202794 (PMC6155441; doi:10.1371/journal.pone.0202794)
Supplement: S1 File — (RTF) [file pone.0202794.s001.rtf]

Time	N	Variable	Mean	SD	Minimum	1st Quartile	Median	3rd Quartile	Maximum	
Baseline	50	C4_0
C6_0
C8_0
C10_0
C11_0
C12_0
C13_0
C14_0
C14_1
C15_0
C15_1
C16_0
C16_1
C17_0
C17_1
C18_0
C18_1n9c
C18_2n6c
C18_3n6
C18_3n3
C20_0
C20_1n9
C20_2
C20_3n6
C21_0
C20_3n3
C20_4n6
C20_5n3
C22_1n9
C22_2
C24_0
C24_1n9
C22_6n3	0.01
0.05
0.14
1.43
0.09
6.11
0.04
6.51
0.17
0.26
0.07
22.30
1.86
0.29
0.17
6.44
30.41
19.62
0.16
1.32
0.13
0.25
0.31
0.05
0.37
0.50
0.35
0.10
0.04
0.04
0.12
0.16
0.10	0.01
0.05
0.12
0.60
0.18
2.30
0.08
2.35
0.06
0.08
0.09
2.80
0.59
0.06
0.04
1.43
4.36
4.11
0.07
0.39
0.07
0.10
0.10
0.09
0.11
0.12
0.79
0.06
0.05
0.02
0.04
0.09
0.10	0.00
0.00
0.01
0.41
0.01
2.12
0.01
3.34
0.07
0.11
0.03
17.24
0.96
0.17
0.07
3.89
19.52
12.64
0.07
0.61
0.07
0.13
0.14
0.01
0.12
0.20
0.05
0.05
0.02
0.01
0.05
0.05
0.02	0.00
0.02
0.05
1.01
0.05
4.69
0.02
5.05
0.12
0.20
0.04
20.64
1.39
0.24
0.14
5.57
27.45
16.24
0.11
0.97
0.10
0.18
0.24
0.02
0.30
0.43
0.13
0.07
0.03
0.02
0.09
0.10
0.04	0.01
0.04
0.13
1.36
0.06
5.66
0.02
5.96
0.17
0.26
0.06
22.24
1.92
0.28
0.17
6.31
30.04
19.96
0.15
1.31
0.12
0.24
0.28
0.03
0.35
0.49
0.21
0.09
0.03
0.03
0.12
0.13
0.08	0.01
0.06
0.21
1.74
0.08
6.77
0.03
7.97
0.21
0.31
0.08
23.46
2.17
0.34
0.20
7.26
33.23
22.30
0.20
1.50
0.14
0.29
0.36
0.04
0.44
0.57
0.32
0.11
0.04
0.04
0.14
0.19
0.12	0.03
0.36
0.78
3.87
1.34
15.26
0.61
15.73
0.33
0.44
0.71
30.88
3.96
0.46
0.29
10.70
42.88
29.15
0.41
2.31
0.54
0.77
0.73
0.69
0.73
0.88
5.72
0.42
0.40
0.11
0.28
0.46
0.61	
Concentrado
	50	C4_0
C6_0
C8_0
C10_0
C11_0
C12_0
C13_0
C14_0
C14_1
C15_0
C15_1
C16_0
C16_1
C17_0
C17_1
C18_0
C18_1n9c
C18_2n6c
C18_3n6
C18_3n3
C20_0
C20_1n9
C20_2
C20_3n6
C21_0
C20_3n3
C20_4n6
C20_5n3
C22_1n9
C22_2
C24_0
C24_1n9
C22_6n3	0.01
0.06
0.18
1.64
0.05
6.91
0.02
6.68
0.17
0.24
0.05
21.46
1.94
0.27
0.17
6.01
30.47
19.88
0.16
1.37
0.11
0.23
0.30
0.03
0.38
0.52
0.16
0.09
0.04
0.03
0.12
0.18
0.06	0.01
0.03
0.08
0.48
0.02
1.87
0.01
2.03
0.06
0.07
0.02
2.23
0.52
0.05
0.03
1.16
3.41
3.68
0.05
0.32
0.03
0.06
0.08
0.01
0.12
0.11
0.08
0.03
0.02
0.01
0.03
0.09
0.03	0.00
0.01
0.03
0.82
0.01
2.98
0.01
3.64
0.08
0.12
0.01
17.01
1.07
0.19
0.08
3.98
25.45
12.43
0.08
0.80
0.07
0.13
0.18
0.00
0.26
0.30
0.03
0.05
0.01
0.01
0.07
0.06
0.01	0.00
0.04
0.12
1.28
0.03
5.46
0.02
5.40
0.13
0.18
0.04
19.86
1.54
0.23
0.15
5.32
27.86
17.81
0.12
1.17
0.09
0.18
0.24
0.02
0.32
0.45
0.10
0.07
0.02
0.02
0.10
0.10
0.03	0.01
0.06
0.18
1.70
0.04
6.70
0.02
6.44
0.16
0.24
0.05
21.53
1.96
0.25
0.17
5.70
29.59
19.98
0.15
1.36
0.10
0.22
0.29
0.03
0.35
0.50
0.14
0.08
0.03
0.03
0.11
0.15
0.05	0.01
0.08
0.23
1.95
0.06
8.31
0.03
7.67
0.22
0.29
0.07
22.52
2.20
0.30
0.19
6.55
31.88
21.79
0.18
1.57
0.12
0.28
0.35
0.03
0.42
0.58
0.21
0.11
0.04
0.04
0.14
0.25
0.07	0.03
0.13
0.39
2.96
0.14
11.38
0.04
12.66
0.36
0.45
0.12
27.26
3.96
0.44
0.25
9.41
42.35
29.42
0.30
2.27
0.22
0.36
0.54
0.04
0.91
0.88
0.37
0.17
0.14
0.08
0.19
0.37
0.15	
3 meses	50	C4_0
C6_0
C8_0
C10_0
C11_0
C12_0
C13_0
C14_0
C14_1
C15_0
C15_1
C16_0
C16_1
C17_0
C17_1
C18_0
C18_1n9c
C18_2n6c
C18_3n6
C18_3n3
C20_0
C20_1n9
C20_2
C20_3n6
C21_0
C20_3n3
C20_4n6
C20_5n3
C22_1n9
C22_2
C24_0
C24_1n9
C22_6n3	0.01
0.06
0.19
1.73
0.04
7.10
0.03
6.82
0.18
0.25
0.06
21.54
1.93
0.27
0.17
5.92
30.55
19.49
0.15
1.34
0.11
0.23
0.30
0.03
0.37
0.52
0.13
0.09
0.03
0.03
0.11
0.17
0.05	0.01
0.03
0.09
0.61
0.02
2.27
0.01
1.95
0.06
0.07
0.02
2.09
0.51
0.05
0.03
1.12
3.56
3.49
0.04
0.32
0.03
0.06
0.08
0.01
0.08
0.10
0.09
0.03
0.01
0.01
0.03
0.09
0.03	0.00
0.02
0.04
0.93
0.01
3.50
0.01
3.62
0.07
0.14
0.02
16.78
1.10
0.17
0.09
3.55
23.80
12.01
0.08
0.69
0.05
0.11
0.18
0.01
0.22
0.30
0.03
0.04
0.01
0.02
0.05
0.04
0.01	0.00
0.04
0.13
1.29
0.02
5.39
0.02
5.38
0.13
0.20
0.04
20.50
1.57
0.24
0.15
5.40
27.67
17.44
0.12
1.13
0.09
0.18
0.25
0.02
0.32
0.46
0.07
0.06
0.03
0.03
0.09
0.11
0.02	0.00
0.05
0.18
1.60
0.04
6.55
0.02
6.47
0.18
0.26
0.06
21.54
1.97
0.26
0.16
5.76
30.72
19.49
0.15
1.33
0.10
0.23
0.28
0.02
0.35
0.50
0.12
0.08
0.03
0.03
0.11
0.14
0.04	0.01
0.07
0.24
2.06
0.05
8.17
0.03
7.80
0.21
0.29
0.07
22.29
2.16
0.30
0.19
6.39
32.83
21.37
0.18
1.51
0.13
0.27
0.35
0.03
0.41
0.57
0.17
0.10
0.03
0.04
0.13
0.25
0.06	0.03
0.19
0.53
3.66
0.13
12.65
0.04
12.88
0.38
0.50
0.14
28.05
4.03
0.46
0.23
9.41
41.47
27.59
0.29
2.21
0.21
0.41
0.51
0.08
0.68
0.83
0.52
0.18
0.07
0.08
0.17
0.44
0.20	
6 meses	50	C4_0
C6_0
C8_0
C10_0
C11_0
C12_0
C13_0
C14_0
C14_1
C15_0
C15_1
C16_0
C16_1
C17_0
C17_1
C18_0
C18_1n9c
C18_2n6c
C18_3n6
C18_3n3
C20_0
C20_1n9
C20_2
C20_3n6
C21_0
C20_3n3
C20_4n6
C20_5n3
C22_1n9
C22_2
C24_0
C24_1n9
C22_6n3	0.00
0.07
0.21
1.77
0.05
7.02
0.19
6.70
0.20
0.26
0.07
21.95
1.99
0.28
0.17
6.00
29.91
19.38
0.17
1.35
0.11
0.23
0.30
0.03
0.38
0.52
0.16
0.09
0.03
0.04
0.12
0.19
0.06	0.00
0.03
0.09
0.51
0.03
1.85
1.03
1.80
0.08
0.08
0.08
2.22
0.51
0.06
0.03
1.09
3.54
3.62
0.06
0.32
0.02
0.06
0.08
0.04
0.10
0.12
0.10
0.04
0.02
0.02
0.03
0.09
0.05	0.00
0.02
0.06
0.95
0.01
3.27
0.01
3.17
0.07
0.13
0.02
18.19
1.13
0.16
0.11
4.22
22.84
12.17
0.09
0.78
0.06
0.14
0.17
0.01
0.25
0.16
0.03
0.04
0.01
0.02
0.06
0.07
0.01	0.00
0.05
0.15
1.46
0.03
6.22
0.02
5.43
0.14
0.20
0.05
20.49
1.66
0.24
0.15
5.41
27.52
17.11
0.12
1.13
0.10
0.19
0.25
0.02
0.32
0.45
0.08
0.06
0.02
0.03
0.09
0.13
0.03	0.00
0.07
0.21
1.77
0.04
7.21
0.02
6.53
0.18
0.24
0.06
22.14
1.94
0.27
0.17
5.77
29.14
19.69
0.15
1.33
0.11
0.23
0.28
0.02
0.37
0.52
0.12
0.07
0.03
0.03
0.11
0.16
0.04	0.01
0.08
0.27
2.03
0.05
8.09
0.03
7.68
0.24
0.31
0.07
23.07
2.18
0.31
0.20
6.71
32.64
21.20
0.19
1.57
0.13
0.28
0.34
0.03
0.43
0.59
0.20
0.11
0.04
0.04
0.14
0.25
0.08	0.01
0.15
0.46
3.02
0.15
10.81
6.64
11.24
0.41
0.48
0.54
27.07
4.04
0.50
0.23
8.69
39.90
26.93
0.42
2.19
0.17
0.37
0.46
0.27
0.79
0.91
0.49
0.20
0.11
0.10
0.19
0.41
0.25	
