# Supplementary material for: Human milk enriched with human milk lyophilisate for feeding very low birth weight preterm infants: A preclinical experimental study focusing on fatty acid profile
Source: PLoS One. 2018 Sep 25;13(9):e0202794. doi: 10.1371/journal.pone.0202794 (PMC6155441; doi:10.1371/journal.pone.0202794)
Supplement: S6 File — (DOCX) [file pone.0202794.s006.docx]

UNIVERSIDADE DE SÃO PAULO

FACULDADE DE MEDICINA DE RIBEIRÃO PRETO

PÓS-GRADUAÇÃO EM SAÚDE DA CRIANÇA E DO ADOLESCENTE

DEPARTAMENTO DE PUERICULTURA E PEDIATRIA

**Perfil Lipídico do Concentrado com Liofilizado de Leite Humano para Alimentação de Recém-nascidos Pré-termo de Muito Baixo Peso**

Mestranda: Vanessa Silva Bomfim

Orientador: Prof. Dr. José Simon Camelo Junior

Ribeirão Preto

Fevereiro de 2017

Contra capa

Resumo

Abreviaturas

Figuras

Tabelas

Índice (revisão de literatura - introdução, justificativa, objetivo, material e métodos, resultados, discussão, perspectiva, cronograma, referencias)

1. INTRODUÇÃO

A nutrição neonatal vem sendo alvo de estudos nas últimas décadas em decorrência, principalmente, da maior sobrevivência de recém-nascidos pré-termo cada vez menores. (1) Essas taxas de sobrevivência e a qualidade de vida de recém-nascidos pré-termo, em especial dos de muito baixo peso, assim considerados os de peso ao nascer menor do que 1.500g têm melhorado significativamente devido aos progressos técnico-científicos incorporados à assistência dessas crianças. Seu bom desenvolvimento também está ligado ao aprimoramento dos métodos de nutrição no período pós-natal imediato. (2)

O terceiro trimestre de gestação é caracterizado por grande desenvolvimento fetal, mediante o aumento de massa óssea, massa muscular e acúmulo de gordura. Os recém-nascidos prematuros perdem uma parte ou todo esse período, conforme a idade gestacional. (3)

Ao nascer prematuramente, muitas vezes, a anatomia do trato digestivo do recém-nascido se completou, porém ainda não houve maturação da sua função, sendo necessário o aporte adequado de nutrientes, em quantidade e qualidade adequadas para o seu desenvolvimento. (4)

O leite humano possui uma composição nutricional balanceada que inclui todos os nutrientes essenciais, além de um grande número dos condicionalmente essenciais e de cerca de 250 ou mais tipos diferentes de fatores bioativos; muitos desses fatores parecem contribuir para o crescimento e desenvolvimento do recém-nascido, bem como para a maturação de seu trato gastrintestinal. Dentre eles destacam-se fatores antimicrobianos, agentes anti-inflamatórios, enzimas digestivas, vários tipos de hormônios, fatores de crescimento e imunomoduladores. (5) Portanto, devido as suas funções fisiológicas, bem como de suas qualidades nutritivas e imunológicas, o leite humano deve fazer parte da dieta do recém-nascido pré-termo de muito baixo peso (RNMBP). (6)

Nas últimas décadas, tem sido dada uma atenção especial à composição e aos aspectos fisiológicos da fração lipídica do leite humano. (7) (8) A porção lipídica do leite materno contribui com 40 a 55% do total de energia ingerida, e provê nutrientes essenciais tais como vitaminas lipossolúveis e ácidos graxos poliinsaturados, incluindo o ácido linoléico da série n-6, (C18:2n-6) e o α-linolênico da série n-3, (C18:3n-3). (9)

Os ácidos graxos que compõem os lipídios do leite são em grande número e pertencentes a dois grupos: o dos ácidos graxos saturados (sem ligações duplas) e o dos ácidos graxos insaturados (com uma ou mais ligações duplas). (10)

Os ácidos graxos saturados e monoinsaturados podem ser sintetizados no organismo, porém os ácidos graxos poliinsaturados, notadamente o linoléico e α-linolênico, por não serem sintetizados pelo organismo constituem-se em ácidos graxos essenciais. Tais ácidos são elementos estruturais necessários à síntese de lipídios de tecidos e têm um papel importante na regulação de vários processos metabólicos, de transporte e excreção. A sua carência conduz a alterações no crescimento, na pele, imunológicas, neurológicas, e sérios transtornos comportamentais. (11)

A qualidade dos lipídios da dieta materna tem influência direta no perfil de ácidos graxos do leite secretado. (12) Uma dieta rica em carboidratos irá favorecer a síntese endógena dos ácidos graxos de cadeia curta e média e uma dieta rica em ácidos graxos poliinsaturados, presente em peixes, leite, carne e ovos, irão determinar maiores níveis destes no leite secretado. (13)

Os ácidos graxos poliinsaturados de cadeia longa (AGPI-CL) no leite materno podem originar-se da ingestão dietética materna, dos estoques maternos e da síntese endógena a partir de seus precursores com 18 carbonos (ácido linoléico e α-linolênico), no fígado, glândula mamária e outros tecidos, produzindo os ácidos araquidônico (ARA, n-6), eicosapentaenoico (EPA, n-3) e docosahexaenóico (DHA, n-3). (7)

Antes do nascimento, todos os ácidos graxos n-6 e n-3 acumulados pelo feto provêm da circulação materna através da transferência placentária, e depois do nascimento originam-se do leite materno quando a amamentação materna for exclusiva. (14) Isso é importante devido ao efeito positivo que esses ácidos graxos representam principalmente para crianças nascidas prematuras e no desenvolvimento do sistema nervoso central e retina. (14) (15) (16).

Os lipídios do cérebro são ricos em AGPI-CL n-3 (especialmente) e n-6 e estes desempenham papel fundamental no crescimento neuronal, transdução de sinais e excitabilidade das membranas neurais, e na expressão de genes que regulam a diferenciação celular e o crescimento. (17)

De fato, o DHA além de exercer efeito positivo no crescimento infantil, é o AGPI com maior presença nos segmentos da retina (cones e bastonetes). Aproximadamente 10% do peso do cérebro e 50% do peso seco são formados por lipídio, sendo metade fosfolipídio. Os fosfolipídios da massa cinzenta do cérebro contêm grandes proporções de DHA e ARA. (11) Altas concentrações de DHA na retina, e de DHA e ARA na massa cinzenta do cérebro sugerem que estes ácidos graxos têm importante função no processo visual e neural. (11) (18) (19).

Portanto, a ingestão dietética adequada desses AGPI-CL ou de seus precursores durante o período perinatal é essencial para o crescimento fetal e neonatal, para o desenvolvimento e para as funções neurológica, comportamental e de aprendizagem. (14)

xxxxxxxxx

A acidez do leite humano pode ser classificada como original e desenvolvida. A original resulta da presença de seus constituintes (micelas de caseína e sais minerais, destacando-se os fosfatos e citratos); e a desenvolvida é consequente ao crescimento bacteriano, da microbiota primária e secundária, que fermentam a lactose produzindo ácido láctico. Com isso há um aumento da osmolaridade e à diminuição da biodisponibilidade do cálcio e do fósforo presentes. (24) (25) (27).

O leite humano possuí mais de 250 substâncias diferentes, dispostas de modo hierarquizado e compartimentalizado, integrando três frações: emulsão, suspensão e solução. (ALMEIDA, 1999) (24) (25)

A fração emulsão congrega os constituintes lipossolúveis; A fração suspensão é constituída por micelas de caseína, formadas por subfrações e a fração solução reúne água e os compostos hidrossolúveis.

Essas três frações apresentam uma relação de proporcionalidade entre si, decorrente do próprio movimento de síntese do leite humano. Dessa maneira, a variação na concentração de um dos constituintes do leite sempre acarreta alteração nos demais, podendo essa relação de proporcionalidade se dar de forma direta ou indireta, dependendo dos constituintes considerados. Por exemplo, os constituintes lipossolúveis, tendem a se relacionar de forma inversamente proporcional com as proteínas do soro (principais imunobiológicos). Tal tendência permite afirmar que quanto maior o conteúdo de gordura, maior será o aporte energético e menor será a concentração de imunobiológicos. (ALMEIDA, 1999) (24) (25)

Estudos acerca da proporcionalidade dos constituintes do leite humano permitiram o estabelecimento da relação matemática entre creme, soro, gordura e conteúdo energético (LUCAS, 1978; LIRA, 2002). O leite humano com conteúdo energético baixo é rico em substâncias protetoras, sobretudo as que se destacam pela proteção química e biológica exercida no trato digestivo do lactente. (24) (25).

**2. JUSTIFICATIVA**

Os RNMBP (<1500g) necessitam de uma maior quantidade de nutrientes para conseguir se desenvolver satisfatoriamente, se comparados aos nascidos a termo. São necessárias maiores quantidades de proteínas, calorias, minerais, micronutrientes e eletrólitos, no entanto devido a sua imaturidade metabólica, tais recém-nascidos não suportam grandes volumes de leite para suprir essas necessidades, necessitando, portanto, de suplementação com misturas multinutrientes com origem na proteína hidrolisada do leite de vaca ou mistura com fórmulas infantis específicas para RN pré-termo.

O leite materno possui tudo que o recém-nascido precisa para um bom desenvolvimento, como diversos nutrientes além de substâncias imunocompetentes (IgA, enzimas, interferon) e moduladores de crescimento. (20).

Atualmente, surgiu a possibilidade de adequação do leite humano acrescido de um concentrado com liofilizado do próprio leite humano. Com isso minimizam-se as reações adversas se comparado a um aditivo de origem heteróloga, capaz de provocar sensibilização e devido a sua alta osmolaridade pode desencadear uma enterocolite necrosante no recém-nascido.

Os ácidos graxos presentes no leite humano são fonte de energia para o neonato e essenciais para o seu desenvolvimento cerebral, pois participam do processo de mielinização e crescimento dos neurônios, atuando também na retina e sendo substância-chave para formação da membrana celular. (21)

Martinez et al. (22) descreveu em um estudo que perdas significativas de nutrientes podem ocorrer na alimentação por sonda, especialmente dos lipídios, devido ao tamanho variado dos glóbulos de gordura, maiores que 4µm que ficam aderidas aos tubos da sonda. Além da perda energética do leite humano ofertado para o recém-nascido, muitos nutrientes importantes são associados com a gordura do leite, por exemplo, as vitaminas lipossolúveis. Portanto, essa perda de gordura é uma real preocupação com possíveis repercussões sobre o crescimento e desenvolvimento dos recém-nascidos. A separação da gordura pode ser evitada pela homogeneização por ultrassom em baixa escala, dando maior estabilidade a esse homogeneizado, minimizando a perda. A utilização de leite humano concentrado com liofilizado de origem humana homogeneizado pode incrementar o teor de gordura do leite ofertado a RNMBP.

É importante conhecer o perfil lipídico do leite que será oferecido para o recém-nascido, em especial a qualidade desses ácidos graxos, principalmente os AGPI-CL, considerados essenciais para o desenvolvimento cerebral dos pré-termos. Os ácidos linoléico e α-linolênico, e seus produtos, ácido araquidônico (ARA) e ácido docosahexaenóico (DHA)/ácido eicosapentaenóico (EPA), respectivamente, influenciam diretamente no desenvolvimento neuronal, acuidade visual e sistema imunológico infantil. (23) Assim, **se justifica** a introdução indispensável desses compostos presentes no leite humano na alimentação do recém-nascido, desde os primeiros dias de vida.

**3. OBJETIVOS**

1. Determinar a quantidade de lipídios totais e o perfil lipídico das amostras de leite humano pasteurizado (Baseline) e do Concentrado com o liofilizado de leite humano pasteurizado no período imediato (CI) e pós-estocagem de 3 (CI 3) e 6 (CI 6) meses, dando ênfase na análise de ácidos graxos essenciais e poliinsaturados. Exemplo: ácido docosahexaenóico, ácido araquidônico, ácido linoléico e α-linoléico.

2. Avaliar a estabilidade nutricional do Concentrado com liofilizado de leite humano no período imediato e pós-estocagem com 3 e 6 meses.

3. Dosar a peroxidação lipídica pós-estocagem.

**4. MATERIAL E MÉTODOS**

Este estudo é parte do *Projeto: Elaboração de um Concentrado com Liofilizado de Leite Humano para Alimentação de Recém-nascidos Pré-termo de Muito Baixo Peso*, financiado pela Fundação Bill e Melinda Gates e CNPq.

Por se tratar de um estudo com material biológico humano, essa pesquisa foi submetida e aprovada pela avaliação do Comitê de Ética em Pesquisa do Hospital das Clínicas da Faculdade de Medicina de Ribeirão Preto – USP (Número do Parecer: 738.080).

O desenvolvimento do projeto de pesquisa foi realizado no Banco de Leite Humano da Faculdade de Medicina de Ribeirão - USP e no Laboratório de Nutrição e Metabolismo do Departamento de Clínica Médica da Faculdade de Medicina de Ribeirão Preto - USP.

**4.1 Coleta de Leite Humano**

As doadoras de leite humano foram esclarecidas quanto à natureza do estudo e convidadas a participar do mesmo. As que aceitaram a participação no projeto assinaram um termo de consentimento livre e esclarecido, passando também por uma triagem clínica e sorológica, como de praxe das doadoras do Banco de Leite. A partir disso, as doadoras foram instruídas em como realizar a massagem e ordenha das mamas para retirada do leite humano em frasco de vidro inerte, esterilizado, fornecido pelo Banco de Leite. Foram coletadas 50 amostras de leite humano maduro com acidez Dornic até 8°D. As amostras que tinham acidez maior que 8°D foram excluídas do estudo.

O leite humano doado foi encaminhado para a sala de processamento do Banco de Leite, no qual foi congelado, para posteriormente serem realizados os procedimentos de controle de qualidade físico-químico, obtenção do Concentrado com o liofilizado de leite humano, pasteurização e controle de qualidade microbiológico.

Todos os procedimentos realizados envolvendo o leite humano seguem a rotina preconizadas pela Rede Brasileira de Bancos de Leite Humano.

**4.1.1 Controle de Qualidade Físico-Químico**

Todas as amostras passaram pelos processos de seleção e classificação preconizados pela Rede Brasileira de Bancos de Leite Humano. A seleção compreende: condições da embalagem, presença de sujidades, cor, off-flavor e acidez Dornic. A classificação compreende a verificação de: período da lactação, acidez Dornic e conteúdo energético – crematócrito. (24) (25) (26)

**4.1.1.1 Processo de seleção**

**4.1.1.1.1 Condições da embalagem**

O leite humano doado foi acondicionado em recipientes de vidro, estéreis, com boca larga e tampa plástica rosqueável. Tem-se com isso o intuito de armazenar o leite em material inerte e inócuo em temperaturas baixas e altas e de não permitir trocas indesejáveis com o produto, assim mantendo o seu valor biológico. (26)

**4.1.1.1.2 Verificação de sujidades**

Foi feita a avaliação se continha algum corpo estranho no leite. São considerados exemplos: pêlos, cabelos, fragmentos de pele, fragmentos de unha, insetos, pedaços de papel, vidro, etc.

**4.1.1.1.3 Verificação da cor**

O leite foi avaliado quanto a sua coloração. São considerados aceitáveis aqueles leites que apresentam coloração que varie do esbranquiçado ao amarelo mais intenso, podendo passar pelo esverdeado e azulado. A coloração do leite pode variar dependendo do momento da ordenha. (24) (25)

**4.1.1.1.4 Verificação de *off-flavor***

*Off-flavor* é a característica organoléptica não conforme com o aroma original do leite humano ordenhado. A sua determinação se configura como importante instrumento de detecção de não-conformidades, sobretudo as que ocorrem do crescimento de microrganismos pertencentes à microbiota secundária do leite, ocasionando alterações físico-químicas em sua composição. (24) (26). Exemplos: rancificação, peixe ou ovo em fase de decomposição, cloro, plástico, remédio.

O frasco de vidro foi agitado em campo de chama, com alto rigor microbiológico e após a remoção da tampa foi inspirado o seu odor.

**4.1.1.1.5 Determinação da Acidez Dornic**

A acidez Dornic do leite humano ordenhado é a acidez titulável expressa em graus Dornic (26).

Para a determinação da acidez titulável do leite humano, foi utilizada a solução titulante que é o hidróxido de sódio 0,1N, também conhecido como Solução Dornic. Cada 0,01ml gasto para neutralizar 1 ml de leite humano ordenhado correspondeu a 1°D (um grau Dornic). (27)

**4.1.1.2 Processo de classificação**

**4.1.1.2.1 Verificação do período de lactação**

O leite humano pode ser classificado em colostro (menos de sete dias após o parto), leite humano de transição (de sete a quatorze dias após o parto) e leite humano maduro (mais de quatorze dias após o parto). (ALMEIDA, 1999) Para o estudo foi utilizado o leite humano maduro.

**4.1.1.2.2 Conteúdo energético – crematócrito**

Crematócrito é a técnica analítica que permite o cálculo estimado do conteúdo energético do leite humano ordenhado. (26)

Foi feita a homogeneização do frasco contendo o leite e posteriormente foi pipetado 1ml de leite para um vidro de penicilina e esses foram dispostos em estante e levado ao banho maria para aquecimento a 40ºC durante 15 minutos. Em seguida foram coletados de forma independente, duas alíquotas de 75 µL de cada amostra com auxílio de microcapilares e vedadas com massa. Os capilares foram centrifugados por 15 minutos em centrifuga de micro-hematócrito. Após a centrifugação são formadas duas colunas: em uma extremidade fica a coluna de creme e na outra a coluna de soro. Com o auxílio de uma régua foi medido o tamanho da coluna (mm) de creme e da coluna total para inserir os resultados nas fórmulas:

**Avaliação do teor de creme:**

Coluna de creme (mm) x 100 / coluna total (mm) = % de creme

**Avaliação do teor de gordura:**

(% creme - 0,59) / 1,46 = % de gordura

**Cálculo do conteúdo energético total:**

(% de creme x 66,8 + 290) = Kcal/L

Em seguida o leite humano passou por algumas etapas visando à obtenção do concentrado com o liofilizado de leite humano, mas antes parte do leite sem nenhuma manipulação, denominada Baseline foi separada para o processo de pasteurização e análise microbiológica e posteriormente para a determinação de lipídios totais e perfil de ácidos graxos.

**4.2 Obtenção do Concentrado com o liofilizado de leite humano**

**4.2.1 Liofilização**

Para a liofilização, 50 ml do leite humano foram transferidos para um recipiente de vidro inerte e estéril e congeladas a -18ºC por 24 hrs. Após esse período as amostras congeladas foram colocadas na câmara de vácuo do liofilizador de bancada (Liofilizador L108®, LioTop).

Após 72 horas, as amostras foram retiradas do liofilizador e mantidas em cadeia de frio para serem reconstituídas com leite humano.

O processo de liofilização promove a desidratação do leite por sublimação. O aumento gradativo da temperatura, com a diminuição da pressão circunvizinha permite que a água congelada presente no leite passe diretamente da fase sólida a gasosa.

**4.2.2 Reconstituição**

As amostras que foram retiradas do liofilizador foram reconstituídas com 75 ml do leite humano (Baseline) da própria doadora e deram origem ao Concentrado Imediato com liofilizado de leite humano (CI). Esses concentrados juntamente com o Baseline passaram pelo processo de pasteurização e controle de qualidade microbiológico.

**4.3 Pasteurização**

O leite humano coletado (Baseline) e o Concentrado Imediato com liofilizado de leite humano (CI) foram pasteurizados a 62,5°C por 30 minutos após o tempo de pré-aquecimento. O processo de pasteurização é descrito a seguir:

O banho-maria foi regulado à temperatura de operação (suficiente para atingir 62,5° no ponto frio) e somente depois de estabilizada a temperatura os frascos (padronizados) contendo o Baseline e o Concentrado Imediato foram postos no banho; O nível de leite no interior da embalagem deve ficar abaixo do nível da água do banho-maria e em função do desprendimento de ar dissolvido no leite humano durante o processo de aquecimento, o rosqueamento das tampas estavam com folga de ¼ de volta (embalagem semifechada);

Assim que a temperatura do leite humano atingiu a marca de 62,5°C (tempo de pré-aquecimento) iniciou-se a marcação do tempo de letalidade térmica (30 minutos); a cada cinco minutos os frascos foram agitados manualmente, sem retirá-los do banho-maria.

Transcorridos os 30 minutos relativos à letalidade térmica, os frascos foram retirados do banho e foi promovido o resfriamento dos mesmos até que o leite humano atingiu uma temperatura igual ou menor a 5°C. (21)

A pasteurização é um tratamento térmico aplicável ao leite humano, que adota como referência a inativação térmica do microrganismo mais termorresistente, a *Coxiella burnetti*. Uma vez observado o binômio temperatura de inativação e tempo de exposição capaz de inativar esse microrganismo, pode-se assegurar que os demais patógenos também estarão termicamente inativados. (28)

**4.4 Controle de qualidade microbiológico**

Para o controle de qualidade microbiológico as amostras de Baseline e CI que foram pasteurizadas passaram pelo procedimento de detecção de coliformes totais.

Foram inoculados duas alíquotas de 1ml cada de Baseline e CI em tubos de 10 ml de caldo bile verde brilhante (50g/L – 5% p/v), com tubos de Durham em seu interior. Após a inoculação e incubação desses tubos a 36 ± 1°C por 24 a 48hrs em estufa bacteriológica, foram analisadas a presença de gás no interior do tubo de Durham, que caracteriza resultado positivo. Se houvessem resultados positivos, por sua vez, essas amostras passariam por uma prova confirmatória, inoculando 0,05 ml de leite em caldo bile verde brilhante (40g/L – 4% p/v) e sendo incubadas pelas mesmas condições do teste inicial. A presença de gás indicando a existência de microrganismos do grupo coliforme confirma que o produto é impróprio para consumo e deve ser descartado. (24) (28) (29)

Após essas etapas realizadas o Concentrado Imediato foi subdividido em outros dois recipientes para o seu armazenamento no período de 3 (C3) e 6 (C6) meses de estocagem, para avaliação da estabilidade nutricional do Concentrado com liofilizado de leite humano. Com todas as amostras foram feitas a análise de lipídios totais (Banco de Leite Humano da Faculdade de Medicina de Ribeirão - USP) e perfil lipídico (Laboratório de Nutrição e Metabolismo do Departamento de Clínica Médica da Faculdade de Medicina de Ribeirão Preto - USP), totalizando 200 amostras (50 de cada grupo).

**4.5 Análise dos lipídios totais**

A quantificação de lipídios totais foi feita pelo Analisador de Leite Humano MIRIS®, após homogeneização com Sonicator MIRIS®, que determinou diretamente o conteúdo nutricional do leite humano a partir de 2ml da amostra.

**4.6 Perfil lipídico**

Para a análise dos ácidos graxos foi feita a extração de gordura através do método de Bligh e Dyer (31) e a metilação com hidróxido de potássio em metanol (KOH/MeOH ) a 0,5 M (reação de derivatização).

Para essa análise foi necessário 0,8 ml de leite colocado em tubos de ensaio de 10 ml com tampa. A essas amostras foram adicionadas 2 ml de clorofórmio e 4 ml de metanol, com posterior agitação de 2 minutos e centrifugação a 3500 rpm por 10 minutos. Todo o sobrenadante foi transferido para outro tubo de 10 ml e adicionado mais 2 ml de clorofórmio e 2 ml de água. Novamente agitado por 1 minuto e centrifugado a 3000 rpm por 8 minutos. A fase superior foi desprezada utilizando uma pipeta tipo Pasteur e a fase remanescente foi filtrada utilizando papel de filtro embebido em clorofórmio. O filtrado foi posto em tubos de 10 ml e seguido para a reação de metilação. Todo o solvente foi evaporado em corrente de N_2_(g). Depois foi acrescentado 1,5 ml de hexano e 0,5 ml de KOH/MeOH. A solução foi agitada por 1 minuto e centrifugada a 3500 rpm por 5 minutos. A camada superior foi transferida para um *vial* de 2 ml e evaporada em corrente de N_2_(g) até um volume aproximado de 250 µL que foi injetado no cromatógrafo gasoso.

Os ésteres metílicos de ácidos graxos foram determinados por cromatografia gasosa utilizando o cromatógrafo gasoso SHIMADZU, GC – 2014 (Shimadzu Europe, Duisburg, Germany), com coluna capilar de polietileno glicol – Supelcowax 10 (30 m de comprimento, 0,25 mm de diâmetro interno; 0,25 µm de espessura do filme; Supelco Inc., Bellefonte, PA). O gás hélio foi utilizado como gás de arraste, com vazão de 1,0 ml/min. O ar sintético foi utilizado para a ionização em chama com detecção a 280°C.

A separação dos ácidos graxos foi feita com um gradiente de temperatura em uma coluna capilar de polietileno glicol. A temperatura inicial da coluna foi de 100 °C, a qual foi mantida por um minuto, logo após, essa temperatura foi acrescida na taxa de 13 °C por minuto a 195°C, mantidos por cinco minutos e, posteriormente, elevada a 240°C, na taxa de 15°C por minutos, onde foi mantida nessa temperatura por 30 minutos. As injeções de 1µl de amostras foram realizadas em modo split. As temperaturas do injetor e do detector foram de 250°C. A identificação dos picos cromatográficos, assim como a determinação da porcentagem dos ácidos graxos presentes nas amostras (Proporção em relação à quantidade total de ácidos graxos identificados) foi feita pela comparação dos tempos de retenção e da área dos picos das amostras com a do padrão externo Supelco 37 Component FAME Mix.

**4.7 Determinação da Atividade Antioxidante Total pelo Método de Redução do Ferro (FRAP)**

O ensaio FRAP (Ferric Reducing Antioxidant Power) avalia a capacidade dos antioxidantes da amostra em reduzir o complexo 2,4,6-tripiridil-s-triazina férrica ([Fe(III)-(TPTZ)2]3+ no complexo ferroso de cor azul intensa [Fe(II)-(TPTZ)2]2+ em meio ácido. Os valores de FRAP são calculados pela medida da absorbância a 593 nm (55). O reagente FRAP foi composto pela mistura de tampão acetato 300mM pH 3,6 com solução TPTZ 10mM em HCl 40mM e solução aquosa de FeCl3 20mM. Utilizou-se 10µl do Concentrado Imediato de 6 meses (C6) aos quais foram acrescentados 300µl de solução FRAP. Em seguida, as amostras foram incubadas à 37ºC por 4 minutos e realizada a leitura em espectrofotômetro.

**4.8 Análise Estatística**

Os dados foram apresentados em valores de média ± desvio padrão, valores mínimos e máximos. Para analisar os diferentes momentos, foi feita análise de variância de medidas repetidas e devido à variabilidade, as variáveis foram consideradas na forma de logaritmo de base exponencial (ln) nas análises. Para as comparações entres os momentos, foram ajustados modelos lineares de efeitos mistos, através do PROC MIXED do software SAS 9.3. Os contrastes ortogonais (comparações múltiplas) foram estimados apenas nos casos em que a hipótese nula (não há diferença entre as médias dos tempos) fosse rejeitada. Em todos os casos o nível de significância foi pré-fixado para p < 0,05.

**5. RESULTADOS**

**5.1 Acidez Dornic e Crematócrito**

Nas 50 amostras de leite humano a média e o desvio padrão da Acidez Dornic foi de 4,34ºD±1,59 e a média e desvio padrão do crematócrito foi de 602,96±139,86 Kcal/L.

**5.2 Controle de qualidade microbiológico**

Todas as amostras analisadas tiveram o teste de qualidade microbiológico negativo.

**5.3 Lipídios totais**

Nas 50 amostras de B a média e o desvio padrão da quantidade de lipídios totais foi de 2,59 ±1,08, no CI foi de 4,03 ±1,44, no C3 foi de 3,68 ±1,34 e no C6 foi de 3,96 ±1,28. Os resultados foram expressos em g/100ml. (Valor p, acréscimo em %)
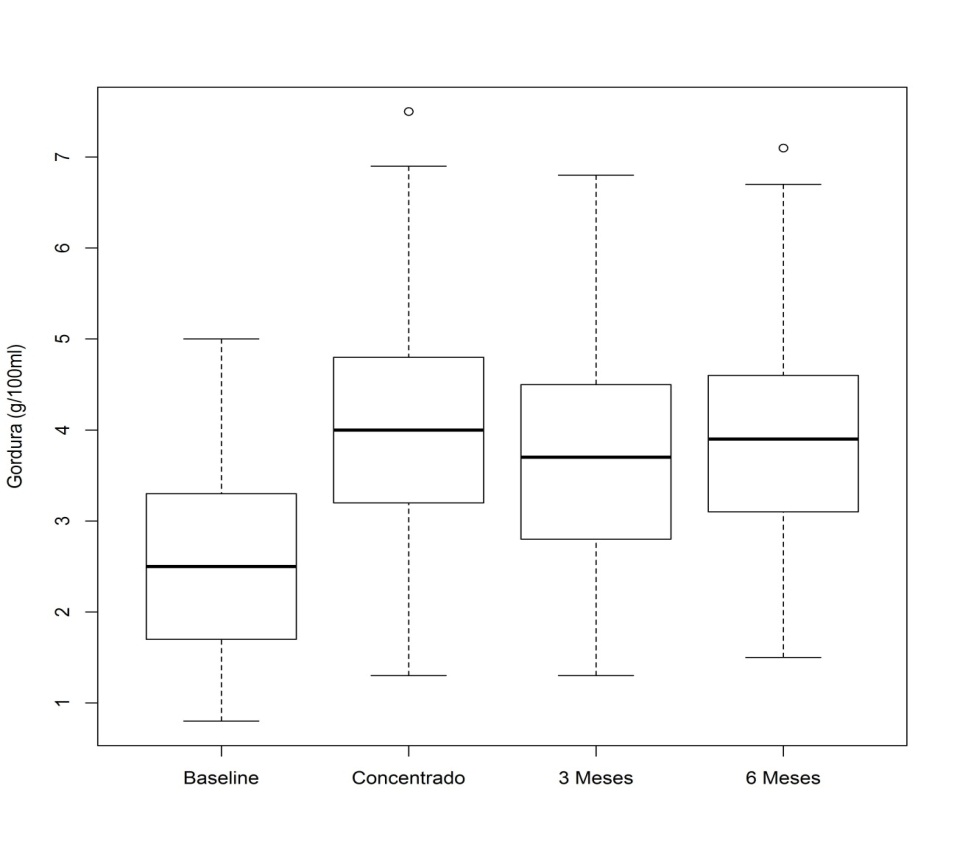


Figura : Box plot da quantidade de lipídios totais.

**5.4 Perfil Lipídico**

Foram identificados 33 ácidos graxos na corrida cromatográfica. A Tabela x descreve a composição de ácidos graxos das amostras do estudo. Os resultados foram expressos em % .

Tabela x: Composição lipídica das amostras B, CI, C3 e C6.

| Ácidos Graxos | B | CI | C3 | C6 | p-valor |
| --- | --- | --- | --- | --- | --- |
| C4:0 (Ácido butírico) | 0,01 ± 0,01 | 0,01 ± 0,01 | 0,01 ± 0,01 | 0 ± 0 | < 0,01* |
| C6:0 (Ácido capróico) | 0,05 ± 0,05 | 0,06 ± 0,03 | 0,06 ± 0,03 | 0,07 ± 0,03 | < 0,01* |
| C8:0 (Ácido caprílico) | 0,14 ± 0,12 | 0,18 ± 0,08 | 0,19 ± 0,09 | 0,21 ± 0,09 | < 0,01* |
| C10:0 (Ácido cáprico) | 1,43 ± 0,60 | 1,64 ± 0,48 | 1,73 ± 0,61 | 1,77 ± 0,51 | < 0,01* |
| C11:0 (Ácido undecílico) | 0,09 ± 0,18 | 0,05 ± 0,02 | 0,04 ± 0,02 | 0,05 ± 0,03 | < 0,01* |
| C12:0 (Ácido láurico) | 6,11 ± 2,30 | 6,91 ± 1,87 | 7,10 ± 2,27 | 7,02 ± 1,85 | < 0,01* |
| C13:0 (Ácido tridecanóico) | 0,04 ± 0,08 | 0,02 ± 0,01 | 0,03 ± 0,01 | 0,19 ± 1,03 | 0,09 |
| C14:0 (Ácido mirístico) | 6,51 ± 2,35 | 6,68 ± 2,03 | 6,82 ± 1,95 | 6,70 ± 1,80 | < 0,01* |
| C14:1 (Ácido miristoléico) | 0,17 ± 0,06 | 0,17 ± 0,06 | 0,18 ± 0,06 | 0,20 ± 0,08 | < 0,01* |
| C15:0 (Ácido pentadecanóico) | 0,26 ± 0,08 | 0,24 ± 0,07 | 0,25 ± 0,07 | 0,26 ± 0,08 | < 0,01* |
| C15:1 (Ácido cis 10-pentadecenóico) | 0,07 ± 0,09 | 0,05 ± 0,02 | 0,06 ± 0,02 | 0,07 ± 0,08 | < 0,01* |
| C16:0 (Ácido palmítico) | 22,30 ± 2,80 | 21,46 ± 2,23 | 21,54 ± 2,09 | 21,95 ± 2,22 | < 0,01* |
| C16:1 (Ácido palmitoléico) | 1,86 ± 0,59 | 1,94 ± 0,52 | 1,93 ± 0,51 | 1,99 ± 0,51 | 0,06 |
| C17:0 (Ácido margárico) | 0,29 ± 0,06 | 0,27 ± 0,05 | 0,27 ± 0,05 | 0,28 ± 0,06 | < 0,01* |
| C17:1 (Ácido cis 10-heptadecanóico) | 0,17 ± 0,04 | 0,17 ± 0,03 | 0,17 ± 0,03 | 0,17 ± 0,03 | 0,38 |
| C18:0 (Ácido esteárico) | 6,44 ± 1,43 | 6,01 ± 1,16 | 5,92 ± 1,12 | 6,00 ± 1,09 | < 0,01* |
| C18:1n9c (Ácido oléico) | 30,41 ± 4,36 | 30,47 ± 3,41 | 30,55 ± 3,56 | 29,91 ± 3,54 | 0,43 |
| C18:2n6c (Ácido linoléico) | 19,62 ± 4,11 | 19,88 ± 3,68 | 19,49 ± 3,49 | 19,38 ± 3,62 | 0,55 |
| C18:3n6 (Ácido γ - linoléico) | 0,16 ± 0,07 | 0,16 ± 0,05 | 0,15 ± 0,04 | 0,17 ± 0,06 | 0,54 |
| C18:3n3 (Ácido α - linolênico) | 1,32 ± 0,39 | 1,37 ± 0,32 | 1,34 ± 0,32 | 1,35 ± 0,32 | 0,16 |
| C20:0 (Ácido araquídico) | 0,13 ± 0,07 | 0,11 ± 0,03 | 0,11 ± 0,03 | 0,11 ± 0,02 | < 0,01* |
| C20:1n9 (Ácido gadoléico) | 0,25 ± 0,10 | 0,23 ± 0,06 | 0,23 ± 0,06 | 0,23 ± 0,06 | 0,37 |
| C20:2 (Ácido 11,14 - eicosadienóico) | 0,31 ± 0,10 | 0,30 ± 0,08 | 0,30 ± 0,08 | 0,30 ± 0,08 | 0,87 |
| C20:3n6 (Ácido dihomo - γ linoléico) | 0,05 ± 0,09 | 0,03 ± 0,01 | 0,03 ± 0,01 | 0,03 ± 0,04 | 0,01* |
| C21:0 (Ácido heneicosanóico) | 0,37 ± 0,11 | 0,38 ± 0,12 | 0,37 ± 0,08 | 0,38 ± 0,10 | 0,43 |
| C20:3n3 (Ácido eicosatrienóico) | 0,50 ± 0,12 | 0,52 ± 0,11 | 0,52 ± 0,10 | 0,52 ± 0,12 | 0,16 |
| C20:4n6 (Ácido araquidônico) | 0,35 ± 0,79 | 0,16 ± 0,08 | 0,13 ± 0,09 | 0,16 ± 0,10 | < 0,01* |
| C20:5n3 (Ácido eicosapentaenóico) | 0,10 ± 0,06 | 0,09 ± 0,03 | 0,09 ± 0,03 | 0,09 ± 0,04 | 0,03* |
| C22:1n9 (Ácido erúcico) | 0,04 ± 0,05 | 0,04 ± 0,02 | 0,03 ± 0,01 | 0,03 ± 0,02 | 0,01* |
| C22:2 (Ácido 13,16 - docosadienóico) | 0,04 ± 0,02 | 0,03 ± 0,01 | 0,03 ± 0,01 | 0,04 ± 0,02 | 0,07 |
| C24:0 (Ácido lignosérico) | 0,12 ± 0,04 | 0,12 ± 0,03 | 0,11 ± 0,03 | 0,12 ± 0,03 | 0,15 |
| C24:1n9 (Ácido lignoceroléico) | 0,16 ± 0,09 | 0,18 ± 0,09 | 0,17 ± 0,09 | 0,19 ± 0,09 | < 0,01* |
| C22:6n-3 (Ácido docosahexaenóico) | 0,10 ± 0,10 | 0,06 ± 0,03 | 0,05 ± 0,03 | 0,06 ± 0,05 | < 0,01* |

Resultados expressos em média ± DP. B - Baseline; CI - Concentrado Imediato; C3 - Concentrado 3 meses; C6 - Concentrado 6 meses. * Houve diferença significativa entre os grupos.

Dentre os ácidos graxos saturados (SFA) o que apresentou maior proporção em relação à quantidade total de ácidos graxos identificados foi o ácido palmítico (C16:0) com 22,30% no B, 21,46% no CI, 21,54% no C3, 21,95% no C6, com diferença estatística entre as médias dos grupos (p<0,01).

No grupo de ácidos graxos monoinsaturados (MFA) o ácido de maior proporção em relação à quantidade total de ácidos graxos identificados foi o ácido oléico (C18:1n-9) com 30,41% no B, 30,47% no CI, 30,55% no C3 e 29,91% no C6, sem diferença estatística entre as médias dos grupos (p=0,43).

No grupo de ácidos graxos poliinsaturados (PUFA) da classe ômega 6 o ácido de maior proporção em relação à quantidade total de ácidos graxos identificados foi o ácido linoléico (C18:2 n-6) com 19,62% no B, 19,88% no CI, 19,49% no C3 e 19,38% no C6, sem diferença estatística entre as médias dos grupos (p=0,55). O principal ácido graxo poliinsaturado de cadeia longa (PUFA) da classe ômega 6 é o ácido araquidônico (C20:4 n-6) que teve uma proporção em relação à quantidade total de ácidos graxos identificados de 0,35% no B, 0,16% no CI, 0,13% no C3 e 0,16% no C6, com diferença estatística entre as médias dos grupos (p<0,01).

No grupo de ácidos graxos poliinsaturados da classe ômega 3 ácido de maior proporção em relação à quantidade total de ácidos graxos identificados foi o ácido α - linolênico (C18:3 n-3) com 1,32% no B, 1,37% no CI, 1,34% no C3 e 1,35% no C6, sem diferença es estatística entre as médias dos grupos (p=0,16). Os principais ácidos graxos poliinsaturados de cadeia longa (PUFA) da classe ômega 3 é o ácido eicosapentaenóico (C20:5 n-3) e o ácido docosahexaenóico (C22:6 n-3) que teve uma proporção em relação à quantidade total de ácidos graxos identificados de 0,10% no B, 0,09% no CI, 0,09% no C3 e 0,09% no C6, e de 0,10% no B, 0,06% no CI, 0,05% no C3 e 0,06% no C6, respectivamente. Ambos apresentaram diferenças estatísticas entre as médias dos grupos: EPA p=0,03 e DHA p<0,01.

Dentre os ácidos graxos saturados (SFA) 12 tiveram diferenças estatísticas entre os tempos. São eles C4:0, C6:0, C8:0, C10:0, C11:0, C12:0, C14:0, C15:0, C16:0, C17:0, C18:0, C20:0.

O ácido butírico C4:0 teve a média do B maior do que a média do C6, média do CI maior do que C3 e C6.

O ácido capróico (C6:0) teve a média do B menor que a média do CI, C3 e C6.

O ácido caprílico (C8:0) teve a média do B menor que a média do CI, C3 e C6.

O ácido cáprico (C10:0) teve a média do B menor que a média do CI, C3 e C6.

O ácido undecílico (C11:0) teve a média B maior do que a média do CI, C3 e C6.

O ácido láurico (C12:0) teve a média do B menor do que a média do CI, C3 e C6.

O ácido mirístico (C14:0) teve a média do B menor do que a média do C3 e C6.

O ácido pentadecanóico (C15:0) teve a média do B maior do que a média do CI, a média do CI menor do que C3 e C6 e a média do C3 menor do que C6.

O ácido palmítico (C16:0) teve a média do B maior do que média do CI e C3 e média do CI maior do que C6.

O ácido margárico (C17:0) teve a média do B maior do que CI e C3, média do CI menor do que C6 e média do C3 menor do que C6.

O ácido esteárico (C18:0) teve a média B maior do que a média do CI, C3 e C6.

O ácido araquídico (C20:0) teve a média B maior do que a média do CI, C3 e C6.

A Tabela x mostra as diferenças estatísticas na comparação entre cada tempo. A diferença das médias está em log (médias geométricas) devido a grande variabilidade dos resultados.

Tabela x:

|  |  |  |  | IC95% | |
| --- | --- | --- | --- | --- | --- |
| SFA | Comparações | Diferença (log) | p-valor | LI | LS |
|  | T1 - T2 | -0,27 | 0,11 | -0,608 | 0,063 |
|  | T1 - T3 | 0,21 | 0,22 | -0,128 | 0,544 |
| C4:0 | T1 - T4 | 0,45 | 0,01* | 0,097 | 0,812 |
|  | T2 - T3 | 0,48 | < 0,01* | 0,145 | 0,816 |
|  | T2 - T4 | 0,73 | < 0,01* | 0,370 | 1,084 |
|  | T3 - T4 | 0,25 | 0,17 | -0,110 | 0,604 |
|  | T1 - T2 | -0,44 | < 0,01* | -0,606 | -0,278 |
|  | T1 - T3 | -0,44 | < 0,01* | -0,608 | -0,279 |
| C6:0 | T1 - T4 | -0,53 | < 0,01* | -0,709 | -0,358 |
|  | T2 - T3 | 0,00 | 0,98 | -0,166 | 0,163 |
|  | T2 - T4 | -0,09 | 0,30 | -0,267 | 0,084 |
|  | T3 - T4 | -0,09 | 0,31 | -0,265 | 0,086 |
|  | T1 - T2 | -0,45 | < 0,01* | -0,610 | -0,284 |
|  | T1 - T3 | -0,50 | < 0,01* | -0,661 | -0,335 |
| C8:0 | T1 - T4 | -0,55 | < 0,01* | -0,725 | -0,377 |
|  | T2 - T3 | -0,05 | 0,54 | -0,214 | 0,112 |
|  | T2 - T4 | -0,10 | 0,24 | -0,278 | 0,070 |
|  | T3 - T4 | -0,05 | 0,55 | -0,227 | 0,121 |
|  | T1 - T2 | -0,18 | < 0,01* | -0,264 | -0,089 |
|  | T1 - T3 | -0,22 | < 0,01* | -0,307 | -0,133 |
| C10:0 | T1 - T4 | -0,23 | < 0,01* | -0,319 | -0,133 |
|  | T2 - T3 | -0,04 | 0,33 | -0,131 | 0,044 |
|  | T2 - T4 | -0,05 | 0,30 | -0,143 | 0,044 |
|  | T3 - T4 | -0,01 | 0,90 | -0,099 | 0,087 |
|  | T1 - T2 | 0,34 | < 0,01* | 0,126 | 0,553 |
|  | T1 - T3 | 0,54 | < 0,01* | 0,325 | 0,753 |
| C11:0 | T1 - T4 | 0,44 | < 0,01* | 0,218 | 0,670 |
|  | T2 - T3 | 0,20 | 0,07 | -0,014 | 0,414 |
|  | T2 - T4 | 0,10 | 0,36 | -0,121 | 0,331 |
|  | T3 - T4 | -0,10 | 0,41 | -0,321 | 0,131 |
|  | T1 - T2 | -0,15 | < 0,01* | -0,217 | -0,079 |
|  | T1 - T3 | -0,17 | < 0,01* | -0,237 | -0,100 |
| C12:0 | T1 - T4 | -0,17 | < 0,01* | -0,240 | -0,094 |
|  | T2 - T3 | -0,02 | 0,56 | -0,089 | 0,048 |
|  | T2 - T4 | -0,02 | 0,61 | -0,092 | 0,054 |
|  | T3 - T4 | 0,00 | 0,97 | -0,072 | 0,075 |
|  | T1 - T2 | -0,04 | 0,13 | -0,087 | 0,012 |
|  | T1 - T3 | -0,07 | < 0,01* | -0,116 | -0,017 |
| C14:0 | T1 - T4 | -0,09 | < 0,01* | -0,141 | -0,036 |
|  | T2 - T3 | -0,03 | 0,26 | -0,078 | 0,021 |
|  | T2 - T4 | -0,05 | 0,06 | -0,104 | 0,002 |
|  | T3 - T4 | -0,02 | 0,41 | -0,075 | 0,031 |
|  | T1 - T2 | 0,08 | < 0,01* | 0,026 | 0,140 |
|  | T1 - T3 | 0,02 | 0,47 | -0,036 | 0,078 |
| C15:0 | T1 - T4 | -0,04 | 0,19 | -0,102 | 0,020 |
|  | T2 - T3 | -0,06 | 0,03* | -0,119 | -0,005 |
|  | T2 - T4 | -0,12 | < 0,01* | -0,184 | -0,062 |
|  | T3 - T4 | -0,06 | 0,04* | -0,123 | -0,001 |
|  | T1 - T2 | 0,04 | < 0,01* | 0,013 | 0,059 |
|  | T1 - T3 | 0,03 | < 0,01* | 0,009 | 0,055 |
| C16:0 | T1 - T4 | 0,01 | 0,45 | -0,015 | 0,034 |
|  | T2 - T3 | 0,00 | 0,71 | -0,027 | 0,018 |
|  | T2 - T4 | -0,03 | 0,03* | -0,051 | -0,003 |
|  | T3 - T4 | -0,02 | 0,07 | -0,047 | 0,002 |
|  | T1 - T2 | 0,07 | < 0,01* | 0,019 | 0,122 |
|  | T1 - T3 | 0,07 | < 0,01* | 0,017 | 0,120 |
| C17:0 | T1 - T4 | 0,01 | 0,78 | -0,047 | 0,062 |
|  | T2 - T3 | 0,00 | 0,95 | -0,053 | 0,050 |
|  | T2 - T4 | -0,06 | 0,03* | -0,117 | -0,008 |
|  | T3 - T4 | -0,06 | 0,03* | -0,116 | -0,006 |
|  | T1 - T2 | 0,06 | < 0,01* | 0,028 | 0,099 |
|  | T1 - T3 | 0,08 | < 0,01* | 0,042 | 0,113 |
| C18:0 | T1 - T4 | 0,05 | < 0,01* | 0,013 | 0,089 |
|  | T2 - T3 | 0,01 | 0,45 | -0,022 | 0,049 |
|  | T2 - T4 | -0,01 | 0,51 | -0,051 | 0,025 |
|  | T3 - T4 | -0,03 | 0,17 | -0,064 | 0,012 |
|  | T1 - T2 | 0,14 | < 0,01* | 0,059 | 0,214 |
|  | T1 - T3 | 0,14 | < 0,01* | 0,059 | 0,213 |
| C20:0 | T1 - T4 | 0,10 | 0,01* | 0,021 | 0,185 |
|  | T2 - T3 | 0,00 | 0,98 | -0,078 | 0,076 |
|  | T2 - T4 | -0,03 | 0,42 | -0,116 | 0,048 |
|  | T3 - T4 | -0,03 | 0,43 | -0,115 | 0,049 |

Resultados expressos em diferença de médias... * Houve diferença estatística nos diferentes tempos.

Dentre os ácidos graxos monosaturados (MFA) 4 tiveram diferenças estatísticas entre os tempos. São eles C14:1, C15:1, C22:1n-9 e C24:1n-9.

O ácido miristoléico (C14:1) teve a média do B menor do que a média do C3 e C6, e média do CI menor do que C6.

O ácido cis 10-pentadecenóico (C15:1) teve a média do B maior do que o CI e a média do CI menor do que C6.

O ácido erúcico (C22:1 n-9) teve a média do B maior do CI, C3 e C6.

O ácido lignoceroléico (C24:1 n-9) teve a média do B menor do que C6.

A Tabela x mostra as diferenças estatísticas na comparação entre cada tempo dos MUFA.

Tabela x:

|  |  |  |  | IC95% | |
| --- | --- | --- | --- | --- | --- |
| MUFA | Comparações | Diferença (log) | p-valor | LI | LS |
|  | T1 - T2 | -0,03 | 0,29 | -0,092 | 0,028 |
|  | T1 - T3 | -0,08 | < 0,01* | -0,143 | -0,024 |
| C14:1 | T1 - T4 | -0,14 | < 0,01* | -0,207 | -0,079 |
|  | T2 - T3 | -0,05 | 0,09 | -0,111 | 0,008 |
|  | T2 - T4 | -0,11 | < 0,01* | -0,175 | -0,047 |
|  | T3 - T4 | -0,06 | 0,07 | -0,123 | 0,005 |
|  | T1 - T2 | 0,20 | < 0,01* | 0,077 | 0,321 |
|  | T1 - T3 | 0,10 | 0,12 | -0,026 | 0,219 |
| C15:1 | T1 - T4 | -0,01 | 0,84 | -0,144 | 0,118 |
|  | T2 - T3 | -0,10 | 0,10 | -0,225 | 0,020 |
|  | T2 - T4 | -0,21 | < 0,01* | -0,343 | -0,081 |
|  | T3 - T4 | -0,11 | 0,10 | -0,240 | 0,021 |
|  | T1 - T2 | 0,13 | 0,04 | 0,006 | 0,247 |
|  | T1 - T3 | 0,19 | < 0,01* | 0,068 | 0,308 |
| C22:1n9 | T1 - T4 | 0,17 | 0,01* | 0,037 | 0,294 |
|  | T2 - T3 | 0,06 | 0,32 | -0,059 | 0,182 |
|  | T2 - T4 | 0,04 | 0,55 | -0,089 | 0,168 |
|  | T3 - T4 | -0,02 | 0,73 | -0,151 | 0,106 |
|  | T1 - T2 | -0,09 | 0,08 | -0,182 | 0,011 |
|  | T1 - T3 | -0,08 | 0,09 | -0,179 | 0,013 |
| C24:1n9 | T1 - T4 | -0,18 | < 0,01* | -0,284 | -0,078 |
|  | T2 - T3 | 0,00 | 0,96 | -0,094 | 0,099 |
|  | T2 - T4 | -0,10 | 0,07 | -0,199 | 0,007 |
|  | T3 - T4 | -0,10 | 0,06 | -0,201 | 0,005 |

Resultados....

Dentre os ácidos graxos poliinsaturados (PUFA) 4 tiveram diferenças estatísticas entre os tempos. São eles C20:3 n-6, C20:4n-6 (ômega 6); C20:5n-3 e C22:6n-3 (ômega 3).

O ácido dihomo - γ linoléico (C20:3n6) teve a média do B maior do que a média de CI, C3 e C6.

O ácido araquidônico (C20:4n6) teve a média do B maior do que a média de CI, C3 e C6 e a média do CI maior do que C3.

O ácido eicosapentaenóico (C20:5n-3) teve a média do B maior do que a média do C3 e C6.

O ácido docosahexaenóico (C22:6n-3) teve a média do B maior do que a média do CI, C3 e C6.

A Tabela x mostra as diferenças estatísticas na comparação entre cada tempo dos PUFA.

Tabela x:

|  |  |  |  | IC95% | |
| --- | --- | --- | --- | --- | --- |
| PUFA | Comparações | Diferença (log) | p-valor | LI | LS |
|  | T1 - T2 | 0,27 | < 0,01* | 0,074 | 0,471 |
|  | T1 - T3 | 0,29 | < 0,01* | 0,092 | 0,489 |
| C20:3n6 | T1 - T4 | 0,22 | 0,04 | 0,008 | 0,429 |
|  | T2 - T3 | 0,02 | 0,85 | -0,180 | 0,217 |
|  | T2 - T4 | -0,05 | 0,61 | -0,264 | 0,157 |
|  | T3 - T4 | -0,07 | 0,50 | -0,283 | 0,138 |
|  | T1 - T2 | 0,42 | < 0,01* | 0,190 | 0,655 |
|  | T1 - T3 | 0,66 | < 0,01* | 0,428 | 0,894 |
| C20:4n6 | T1 - T4 | 0,47 | < 0,01* | 0,223 | 0,716 |
| (ARA) | T2 - T3 | 0,24 | 0,04 | 0,006 | 0,471 |
|  | T2 - T4 | 0,05 | 0,71 | -0,200 | 0,294 |
|  | T3 - T4 | -0,19 | 0,13 | -0,438 | 0,055 |
|  | T1 - T2 | 0,06 | 0,11 | -0,015 | 0,143 |
|  | T1 - T3 | 0,11 | < 0,01* | 0,033 | 0,191 |
| C20:5n3 | T1 - T4 | 0,09 | 0,03 | 0,008 | 0,176 |
| (EPA) | T2 - T3 | 0,05 | 0,23 | -0,031 | 0,127 |
|  | T2 - T4 | 0,03 | 0,51 | -0,056 | 0,113 |
|  | T3 - T4 | -0,02 | 0,64 | -0,104 | 0,064 |
|  | T1 - T2 | 0,42 | < 0,01* | 0,184 | 0,652 |
|  | T1 - T3 | 0,63 | < 0,01* | 0,401 | 0,868 |
| C22:6n3 | T1 - T4 | 0,45 | < 0,01* | 0,204 | 0,699 |
| (DHA) | T2 - T3 | 0,22 | 0,07 | -0,017 | 0,450 |
|  | T2 - T4 | 0,03 | 0,79 | -0,214 | 0,282 |
|  | T3 - T4 | -0,18 | 0,15 | -0,431 | 0,065 |

Resultados...

**5.5 Peroxidação lipídica**

**6. DISCUSSÃO**

**7. CRONOGRAMA**

| **Atividades** | **1º Semestre** | **2º Semestre** | **3º Semestre** | **4º Semestre** | **5º Semestre** | **6º Semestre** |
| --- | --- | --- | --- | --- | --- | --- |
| **Revisão de Literatura** |  |  |  |  |  |  |
| **Acompanhamento da rotina do Banco de Leite - FMRP-USP** |  |  |  |  |  |  |
| **Treinamento da técnica** |  |  |  |  |  |  |
| **Coleta das Amostras** |  |  |  |  |  |  |
| **Processamento das Amostras** |  |  |  |  |  |  |
| **Determinação de ácidos graxos** |  |  |  |  |  |  |
| **Análise de dados e estatística** |  |  |  |  |  |  |
| **Confecção de artigos para publicação** |  |  |  |  |  |  |

**REFERÊNCIAS**

20- COSTA, André Gustavo Vasconcelos; SABARENSE, Céphora Maria. Modulação e composição de ácidos graxos do leite humano.**Rev. Nutr.**,  Campinas,  v. 23, n. 3, p. 445-457, June 2010.

21- MARTINEZ, F.E.; CAMELO JR, J.S. Alimentação do recém-nascido pré-termo. J Pediatr. Rio de Janeiro. 2001; 77(suppl1): 32-40.

22- MARTINEZ, F. E.; DESAI, I. D.; DAVIDSON, A. G. F.; NAKAI, S.; RADCLIFFE, A. Ultrasonic Homogenization of Expressed Human Milk to Prevent Fat Loss During Tube Feeding. Journal of Pediatric Gastroenterology & Nutrition. 1987 Jul-Aug;6(4):593-7.

23-NISHIMURA RY, CASTRO GS, JUNIOR AA, SARTORELLI DS. Breast milk fatty acid composition of women living far from the coastal area in Brazil. J Pediatr (Rio J). 2013;89:263-8.

24- FIOCRUZ (FUNDAÇÃO OSWALDO CRUZ). Programa Nacional de Qualidade em Bancos de Leite Humano. Rio de Janeiro, 2003.

25- SILVA, V.G. Normas técnicas para banco de leite humano: uma proposta para subsidiar a construção para Boas Práticas. Tese (Doutorado em Saúde da Mulher e da Criança) – Instituto Fernandes Figueira/Fundação Oswaldo Cruz, Rio de Janeiro, 2004.

26- BRASIL, Agência Nacional de Vigilância Sanitária. Resolução RDC n° 171, de 4 de setembro de 2006. Dispõe sobre Regulamento Técnico para o funcionamento de Bancos de Leite Humano. Diário Oficial da União, Brasília, DF, 5 de set.2006

27- ALMEIDA, J.A G.; NOVAK, F.R.; SANDOVAL, M.H. Recomengaciones técnicas para los bancos de leche humana II: control de calidad. *Archivos Venezolanos de Puericultura y Pediatría,* Caracas, v.61, n.1, p.12-15. enero/marzo, 1998.

ALMEIDA, J.A.G. *Amamentação:* um híbrido natureza-cultura. Rio de Janeiro: Fiocruz, 1999

28- BRASIL. Ministério da Saúde. Recomendações técnicas para o funcionamento de bancos de leite humano. 4.ed. Brasília, 2001.48 p. (Série A. Normas e Manuais Técnicos, n. 117).

29- NOVAK, F. R.; ALMEIDA, J.A. G. Teste alternativo para a detecção de coliformes em leite humano ordenhado. Jornal de Pediatria, Rio de Janeiro, v.78, n.3, p. 587-591, maio/ jun. 2002.

31- BLIGH EG, DYER WJ. A rapid method of total lipid extraction and purification. Can J Biochem Physiol. 1959;37:911-7.

55. Benzie IF, Strain JJ. The ferric reducing ability of plasma (FRAP) as a measure of "antioxidant power": the FRAP assay. Anal Biochem. 1996 Jul 15;239(1):70-6. PubMed PMID: 8660627. Epub 1996/07/15. eng.
