# Supplementary figures and images for: Human milk enriched with human milk lyophilisate for feeding very low birth weight preterm infants: A preclinical experimental study focusing on fatty acid profile
Source: PLoS One. 2018 Sep 25;13(9):e0202794. doi: 10.1371/journal.pone.0202794 (PMC6155441; doi:10.1371/journal.pone.0202794)

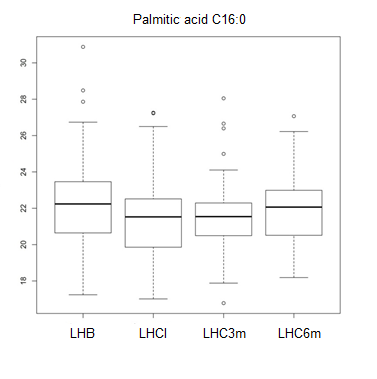

Supplement: S1 Fig — (PNG) [file pone.0202794.s007.png]

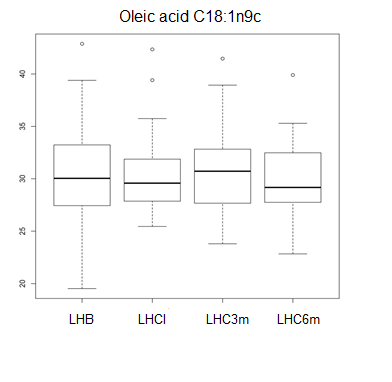

Supplement: S2 Fig — (PNG) [file pone.0202794.s008.png]

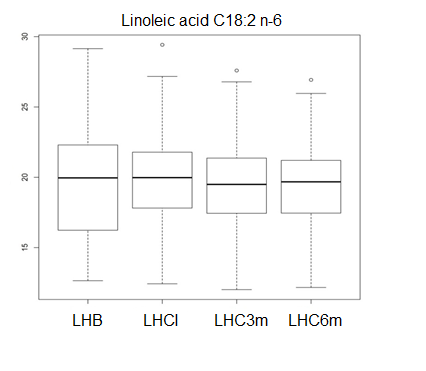

Supplement: S3 Fig — (PNG) [file pone.0202794.s009.png]

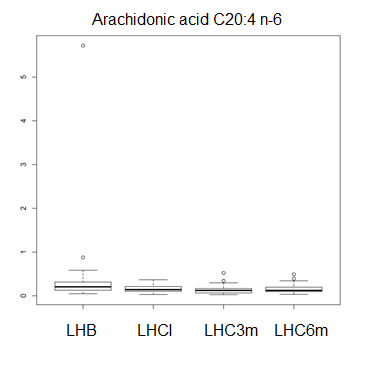

Supplement: S4 Fig — (PNG) [file pone.0202794.s010.png]

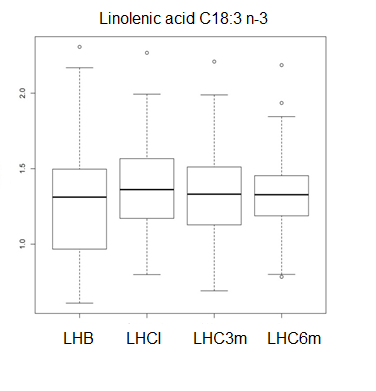

Supplement: S5 Fig — (PNG) [file pone.0202794.s011.png]

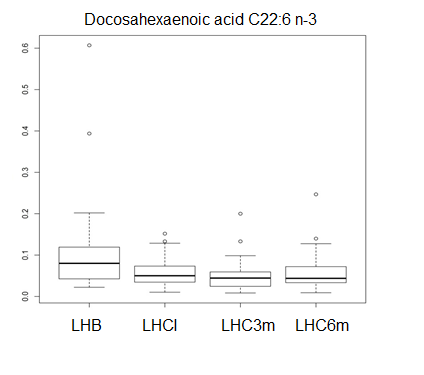

Supplement: S6 Fig — (PNG) [file pone.0202794.s012.png]
