# Supplementary material for: Human milk enriched with human milk lyophilisate for feeding very low birth weight preterm infants: A preclinical experimental study focusing on fatty acid profile
Source: PLoS One. 2018 Sep 25;13(9):e0202794. doi: 10.1371/journal.pone.0202794 (PMC6155441; doi:10.1371/journal.pone.0202794)
Supplement: S1 Table — (PDF) [file pone.0202794.s013.pdf]

Table 1: Comparative values of saturated fatty acids (SFA) at different analysis times

SFA	Comparisons	Differences (log)	p-value	CI95%	
				IL	UL
C4:0	T1 - T2	-0,273	0,11	-0,606	0,061
	T1 - T3	0,208	0,22	-0,126	0,542
	T1 - T4	0,362	0,03*	0,028	0,695
	T2 - T3	0,480	< 0,01*	0,147	0,814
	T2 - T4	0,634	< 0,01*	0,301	0,968
	T3 - T4	0,154	0,36	-0,180	0,488
C6:0	T1 - T2	-0,442	< 0,01*	-0,602	-0,282
	T1 - T3	-0,444	< 0,01*	-0,604	-0,284
	T1 - T4	-0,544	< 0,01*	-0,704	-0,384
	T2 - T3	-0,002	0,98	-0,162	0,158
	T2 - T4	-0,102	0,21	-0,262	0,058
	T3 - T4	-0,101	0,22	-0,261	0,060
C8:0	T1 - T2	-0,447	< 0,01*	-0,606	-0,288
	T1 - T3	-0,498	< 0,01*	-0,657	-0,339
	T1 - T4	-0,549	< 0,01*	-0,708	-0,390
	T2 - T3	-0,051	0,53	-0,210	0,108
	T2 - T4	-0,102	0,21	-0,261	0,057
	T3 - T4	-0,051	0,53	-0,210	0,108
C10:0	T1 - T2	-0,177	< 0,01*	-0,262	-0,091
	T1 - T3	-0,220	< 0,01*	-0,305	-0,135
	T1 - T4	-0,221	< 0,01*	-0,306	-0,136
	T2 - T3	-0,043	0,32	-0,128	0,042
	T2 - T4	-0,045	0,30	-0,130	0,041
	T3 - T4	-0,001	0,98	-0,086	0,084
C11:0	T1 - T2	0,339	< 0,01*	0,126	0,553
	T1 - T3	0,539	< 0,01*	0,325	0,753
	T1 - T4	0,468	< 0,01*	0,254	0,682
	T2 - T3	0,200	0,07	-0,014	0,414
	T2 - T4	0,129	0,24	-0,085	0,342
	T3 - T4	-0,071	0,51	-0,285	0,143
C12:0	T1 - T2	-0,148	< 0,01*	-0,215	-0,081
	T1 - T3	-0,168	< 0,01*	-0,235	-0,101
	T1 - T4	-0,166	< 0,01*	-0,233	-0,100
	T2 - T3	-0,020	0,55	-0,087	0,046
	T2 - T4	-0,019	0,59	-0,085	0,048
	T3 - T4	0,002	0,96	-0,065	0,069
C14:0	T1 - T2	-0,038	0,12	-0,086	0,010
	T1 - T3	-0,066	< 0,01*	-0,114	-0,018
	T1 - T4	-0,082	< 0,01*	-0,131	-0,034

	T2 - T3	-0,028	0,25	-0,077	0,020
	T2 - T4	-0,044	0,07	-0,093	0,004
	T3 - T4	-0,016	0,51	-0,064	0,032
C15:0	T1 - T2	0,083	< 0,01*	0,027	0,139
	T1 - T3	0,021	0,46	-0,035	0,077
	T1 - T4	-0,023	0,41	-0,079	0,033
	T2 - T3	-0,062	0,03*	-0,118	-0,006
	T2 - T4	-0,106	< 0,01*	-0,162	-0,050
	T3 - T4	-0,044	0,12	-0,100	0,012
C16:0	T1 - T2	0,036	< 0,01*	0,014	0,058
	T1 - T3	0,032	< 0,01*	0,010	0,054
	T1 - T4	0,013	0,26	-0,009	0,035
	T2 - T3	-0,004	0,70	-0,026	0,018
	T2 - T4	-0,023	0,04*	-0,046	-0,001
	T3 - T4	-0,019	0,09	-0,041	0,003
C17:0	T1 - T2	0,070	< 0,01*	0,020	0,120
	T1 - T3	0,068	< 0,01*	0,018	0,119
	T1 - T4	0,015	0,57	-0,036	0,065
	T2 - T3	-0,002	0,94	-0,052	0,048
	T2 - T4	-0,056	0,03*	-0,106	-0,005
	T3 - T4	-0,054	0,04*	-0,104	-0,004
C18:0	T1 - T2	0,064	< 0,01*	0,029	0,098
	T1 - T3	0,077	< 0,01*	0,043	0,112
	T1 - T4	0,053	< 0,01*	0,019	0,088
	T2 - T3	0,014	0,44	-0,021	0,048
	T2 - T4	-0,010	0,56	-0,045	0,024
	T3 - T4	-0,024	0,18	-0,059	0,011
C20:0	T1 - T2	0,137	< 0,01*	0,062	0,212
	T1 - T3	0,136	< 0,01*	0,061	0,211
	T1 - T4	0,098	< 0,01*	0,023	0,173
	T2 - T3	-0,001	0,98	-0,076	0,074
	T2 - T4	-0,039	0,31	-0,114	0,036
	T3 - T4	-0,038	0,32	-0,113	0,037

Results expressed by the difference of the geometric means in the different groups. * There was statistical difference. T1: Human Milk Baseline (time 1); T2: Concentrated with human milk lyophilized in the immediate period (time 2); T3: Concentrated with human milk lyophilized at 3 months (time 3); T4: Concentrated with human milk lyophilized at 6 months (time 4).
