# Supplementary material for: Human milk enriched with human milk lyophilisate for feeding very low birth weight preterm infants: A preclinical experimental study focusing on fatty acid profile
Source: PLoS One. 2018 Sep 25;13(9):e0202794. doi: 10.1371/journal.pone.0202794 (PMC6155441; doi:10.1371/journal.pone.0202794)
Supplement: S2 Table — (PDF) [file pone.0202794.s014.pdf]

Table 2: Comparative values of monounsaturated fatty acids (MUFA) at different times of analysis.

| MUFA | Comparisons | Differences (log) | p-value | CI95% IL | CI95% UL |
|---|---|---|---|---|---|
| C14:1 | T1 - T2 | -0,032 | 0,28 | -0,090 | 0,027 |
| | T1 - T3 | -0,084 | < 0,01* | -0,142 | -0,025 |
| | T1 - T4 | -0,125 | < 0,01* | -0,183 | -0,066 |
| | T2 - T3 | -0,052 | 0,08 | -0,110 | 0,007 |
| | T2 - T4 | -0,093 | < 0,01* | -0,152 | -0,034 |
| | T3 - T4 | -0,041 | 0,17 | -0,100 | 0,017 |
| C15:1 | T1 - T2 | 0,199 | < 0,01* | 0,078 | 0,320 |
| | T1 - T3 | 0,096 | 0,12 | -0,024 | 0,217 |
| | T1 - T4 | 0,024 | 0,70 | -0,097 | 0,144 |
| | T2 - T3 | -0,103 | 0,09 | -0,223 | 0,018 |
| | T2 - T4 | -0,175 | < 0,01* | -0,296 | -0,055 |
| | T3 - T4 | -0,073 | 0,24 | -0,193 | 0,048 |
| C22:1n9 | T1 - T2 | 0,127 | 0,03* | 0,009 | 0,244 |
| | T1 - T3 | 0,188 | < 0,01* | 0,070 | 0,305 |
| | T1 - T4 | 0,174 | < 0,01* | 0,057 | 0,291 |
| | T2 - T3 | 0,061 | 0,30 | -0,056 | 0,179 |
| | T2 - T4 | 0,048 | 0,43 | -0,070 | 0,165 |
| | T3 - T4 | -0,014 | 0,82 | -0,131 | 0,104 |
| C24:1n9 | T1 - T2 | -0,085 | 0,08 | -0,180 | 0,010 |
| | T1 - T3 | -0,083 | 0,09 | -0,178 | 0,012 |
| | T1 - T4 | -0,166 | < 0,01* | -0,261 | -0,071 |
| | T2 - T3 | 0,002 | 0,96 | -0,092 | 0,097 |
| | T2 - T4 | -0,081 | 0,10 | -0,176 | 0,014 |
| | T3 - T4 | -0,083 | 0,09 | -0,178 | 0,012 |

Results expressed by the difference of the geometric means in the different groups. * There was statistical difference. T1: Human Milk Baseline (time 1); T2: Concentrated with human milk lyophilized in the immediate period (time 2); T3: Concentrated with human milk lyophilized at 3 months (time 3); T4: Concentrated with human milk lyophilized at 6 months (time 4).
