# Supplementary material for: Human milk enriched with human milk lyophilisate for feeding very low birth weight preterm infants: A preclinical experimental study focusing on fatty acid profile
Source: PLoS One. 2018 Sep 25;13(9):e0202794. doi: 10.1371/journal.pone.0202794 (PMC6155441; doi:10.1371/journal.pone.0202794)
Supplement: S3 Table — (PDF) [file pone.0202794.s015.pdf]

Table 3: Comparative values of polyunsaturated fatty acids (PUFA) at different analysis times.

PUFA	Comparisons	Differences (log)	p-value	CI95%	
				IL	UL
C20:3n6	T1 - T2	0,272	< 0,01*	0,078	0,467
	T1 - T3	0,291	< 0,01*	0,096	0,485
	T1 - T4	0,258	< 0,01*	0,064	0,453
	T2 - T3	0,019	0,85	-0,176	0,213
	T2 - T4	-0,014	0,89	-0,209	0,181
	T3 - T4	-0,033	0,74	-0,227	0,162
C20:4n6 (ARA)	T1 - T2	0,423	< 0,01*	0,194	0,652
	T1 - T3	0,661	< 0,01*	0,432	0,890
	T1 - T4	0,493	< 0,01*	0,264	0,722
	T2 - T3	0,238	0,04*	0,009	0,467
	T2 - T4	0,070	0,54	-0,158	0,299
	T3 - T4	-0,168	0,15	-0,397	0,061
C20:5n3 (EPA)	T1 - T2	0,064	0,11	-0,014	0,142
	T1 - T3	0,112	< 0,01*	0,035	0,190
	T1 - T4	0,117	< 0,01*	0,039	0,194
	T2 - T3	0,048	0,22	-0,029	0,126
	T2 - T4	0,053	0,18	-0,025	0,130
	T3 - T4	0,004	0,91	-0,073	0,082
C22:6n3 (DHA)	T1 - T2	0,418	< 0,01*	0,188	0,648
	T1 - T3	0,634	< 0,01*	0,404	0,864
	T1 - T4	0,477	< 0,01*	0,247	0,707
	T2 - T3	0,216	0,07	-0,014	0,447
	T2 - T4	0,059	0,61	-0,171	0,289
	T3 - T4	-0,157	0,18	-0,387	0,073

Results expressed by the difference of the geometric means in the different groups. * There was statistical difference. T1: Human Milk Baseline (time 1); T2: Concentrated with human milk lyophilized in the immediate period (time 2); T3: Concentrated with human milk lyophilized at 3 months (time 3); T4: Concentrated with human milk lyophilized at 6 months (time 4).
